# Evaluation of the Psychometric Properties of the Social Communication Questionnaire in Rural Kenya

**DOI:** 10.1007/s10803-024-06380-9

**Published:** 2024-05-30

**Authors:** Patricia Kipkemoi, Jeanne E. Savage, Joseph Gona, Kenneth Rimba, Martha Kombe, Paul Mwangi, Collins Kipkoech, Danielle Posthuma, Charles R. J. C. Newton, Amina Abubakar

**Affiliations:** 1 Neuroscience Unit, KEMRI-Wellcome Trust Research Programme, P.O Box 230-80108, Kilifi, Kenya; 2 Complex Trait Genetics Department, Center for Neurogenomics and Cognitive Research (CNCR), Vrije Universiteit Amsterdam, Amsterdam, Netherlands; 3 Department of Child and Adolescent Psychology and Psychiatry, Complex Trait Genetics, Amsterdam University Medical Centres, Vrije Universiteit Amsterdam, Amsterdam Neuroscience, Amsterdam, The Netherlands; 4 Department of Psychiatry, University of Oxford, Warneford Hospital, Warneford Ln, Oxford, OX3 7JX UK; 5 Department of Public Health, Pwani University, P.O. BOX, Kilifi, 195-80108 Kenya; 6 Institute for Human Development, Aga Khan University, P.O. BOX, Nairobi, 30270-00100 Kenya

**Keywords:** Autism, Screening, Kenya, Psychometrics

## Abstract

Children can be reliably diagnosed with autism as early as 3 years of age, and early interventions are initiated. There is often a significant gap between the age of onset of symptoms (2–3 years) and diagnosis (8–10 years) in Africa. We conducted a study to validate the Social Communication Questionnaire (SCQ) as a screening instrument in a rural setting in Kenya. The study was conducted along the Kenyan Coast. Study participants included 172 children with a neurodevelopmental disorder (NDD) diagnosis (84 of which were autism) and 112 controls. Internal consistency was evaluated through the use of Cronbach’s alpha, confirmatory factor analysis (CFA) with maximum likelihood procedure to assess the conceptual model for the SCQ. Additionally, the sensitivity and specificity of cut-off scores using ROC analysis and item difficulties and discrimination quality using an IRT framework were also assessed. Factor analysis revealed an adequate fitting model for the three-factor DSM-IV-TR (root mean squared error of approximation (RMSEA) = 0.050; Comparative Fit Index (CFI) = 0.974; Tucker-Lewis Index (TLI) = 0.973) and two-factor DSM-5 factor structure (RMSEA = 0.050; CFI = 0.972; TLI = 0.974). The reliability coefficient alphas for the whole group for all items (Cronbach’s α = 0.90) and all three domains (Cronbach’s α = 0.68–0.84) were acceptable to excellent. The recommended cut-off score of 15 yielded 72% sensitivity and 100% specificity in the ASD group compared to the typically developing group. We provide early evidence of the adequate factor structure and good internal consistency of the SCQ. We also note that the recommended cut-off yielded sufficient predictive validity.

**Supplementary Information:**

The online version contains supplementary material available at 10.1007/s10803-024-06380-9.

## Introduction

Autism is a neurodevelopmental condition with differences in reciprocal social interaction, language and communication challenges, and restricted, repetitive behaviours and interests as key characteristics (APA, 2022). Valid screening and diagnostic tools are critical to aid the identification and study of autism, its associated symptomatology, and related disorders. Children can be reliably diagnosed with autism as early as 2 years of age, and early interventions can be initiated thereafter (Guthrie et al., 2013; Lord, 1995; Matson & Horovitz, 2010; Moore & Goodson, 2003). However, in Africa, there is often a significant gap between the age of onset of symptoms (2–3 years) and diagnosis (8–10 years) (Kauchali & Davidson, 2006; Bakare & Munir, 2011). The prevalence estimate of autism in Kenya and much of Africa is not well known, hindering proper care and intervention planning (Abubakar et al., 2016; Franz et al., 2017; Ruparelia et al., 2016). Early intervention has been shown to positively impact the developmental trajectory (Landa & Kalb, 2012; Matson & Smith, 2008). Due to diagnostic delays, interventions that could improve verbal and non-verbal communication are not carried out early.

Regardless of a child’s age, screening and diagnostic efforts require appropriate measures that accurately and reliably identify cases with the condition in question (Schanding et al., 2012). The Social Communication Questionnaire (SCQ; Appendix 1) (Rutter et al., 2003), formerly the Autism Screening Questionnaire, is a parent-report questionnaire developed based on the established parental interview, the autism diagnostic interview (ADI) (Lord et al., 1994) and DSM-IV and is widely recognised as an autism screening tool. It has also been shown to be a valid instrument for diagnosing autism in children from 2 years onwards (Berument et al., 1999). The SCQ has been used in research and clinical settings, mainly in high-income settings (Chandler et al., 2007; Eaves et al., 2006; Gau et al., 2011; Mulligan et al., 2008). It takes approximately 15 min to answer and is a cost-effective way to determine whether an individual should be referred for a complete diagnostic evaluation.

Agreement between the SCQ and other diagnostic instruments seems to vary and depends on how the diagnosis was defined. Diagnostic accuracy is improved when clinical judgement is used alongside standardised observational and parental report measures (Lord et al., 2006). The Autism Diagnostic Observation Schedule (ADOS) (Lord et al., 2000) is considered one of the “gold standard’ assessments for an autism diagnosis. It is a standardised observational assessment comprising ten tasks and is organised into four separate modules based on the age and expressive language of the child. Comparison of the SCQ to the ADOS has been mixed: in one study, there was moderate to a reasonable agreement (Chandler et al., 2007), but another study did not find good agreement (Bishop & Norbury, 2002). In addition to studies into the SCQ’s external validity, a few studies (as reviewed by Wei et al., 2015) have also evaluated the internal validity of the SCQ using an item response theory approach. One of the limitations of the SCQ mentioned in the literature is its summing of raw scores to get the total scores. For this summation, all items are assumed to contribute equally to the total scale score; however, this may not be the case as different items may be better or poorer indices of autism (Barnard-Brak et al., 2016), especially across different cultural contexts. Evaluating its item-level characteristics is also imperative as it would further inform its psychometric properties and future practical use. Studies have found acceptable to excellent internal consistency for the SCQ (Wei et al., 2015; Zarokanellou et al., 2017). These studies also found that 85% of the items demonstrate high discrimination. However, these studies have primarily been carried out in high-income populations, so more evidence on item analysis from diverse cultural settings is crucial.

In much of Africa, there are still difficulties in identifying autism due to limited resources in education and healthcare facilities and limited culturally appropriate screening and diagnostic tools (Abubakar et al., 2016). Diagnosing autism requires substantial resources for clinician-dependent assessment tools such as the ADI-R and the ADOS, particularly in lower- and middle-income countries, where we have a limited number of mental health care providers (Abubakar et al., 2016). At least five validation studies of the SCQ have been done in Africa. In the first, an evaluation of the psychometric properties of the English, Afrikaans and IsiXhosa adaptations of the SCQ in the Western Cape in South Africa, the author found acceptable levels of internal consistency in the three languages evaluated in the study (Bozalek, 2013) and concluded that the SCQ appears to capture autism symptomatology. The second study based in Bamako, Mali validates the SCQ alongside the modified checklist for autism in toddlers-Revised (M-CHAT-R) and found a specificity of 71% and specificity of 72% and found that the SCQ would be a useful tool in autism screening (Sangare et al., 2019). The third study based in Uganda found that the SCQ and the social responsive scale, second edition (SRS-2), both showed adequate internal consistency and validity, thereby being useful in distinguishing autistic children and typically developing children (Awadu, 2021). A fourth validation study of the SCQ in Tanzania also found excellent internal consistency and test–retest reliability (Ruparelia, 2021). A fifth study in Nigeria reported good internal consistency, discriminative and convergent validity (Nwokolo et al., 2024). Psychometric studies of the SCQ have, however, not been done in Kenya. False positives involve costly further investigation and parental anxiety. False negatives may deprive children of clinical and educational resources or place the burden of provision entirely on parents (Charman et al., 2016). It is, therefore, crucial that the efforts into valid and reliable screeners in the autism evaluation process are continued and sustained.

Given these gaps in autism research in Africa, we conducted a study to develop a screening tool for identifying autism in children in Kenya based on the SCQ lifetime scale. This tool utilises caregiver/teacher assessment of the child’s behaviour to make it easily applicable across many settings at a low cost in this context. We specifically aim to examine the reliability of the SCQ, model the item-level characteristics of the SCQ, confirm the factorial structure of the SCQ evaluated against the three-factor DSM-IV and the two-factor DSM-5 criteria, and model the item-level characteristics of the SCQ.

## Methods

### Study Setting and Participants

This study was nested in a broader project called the Autism Study, which aimed to understand the experiences of children with autism in Kilifi, identify available services and start developing identification and support systems for autistic children, validating measures of autism. The participants were recruited from mainstream schools, special needs units and special needs schools in Kilifi and Mombasa counties in Kenya. The sample included 268 children; 167 were noted by a disability assessor (J.G) to have a neurodevelopmental concern from teacher and caregiver reports, and 101 were reported to be typically developing. We further categorised participants into the autism group following a positive autism diagnosis on the ADOS (version 2) (Lord et al., 2012) or the DSM-IV-TR clinical confirmation diagnoses from ADOS videos (Autism subgroup; *n* = 78). We then had a category of children with neurodevelopmental disabilities as reported from reported developmental concerns and a negative autism diagnosis from the ADOS and the DSM-IV-TR clinical confirmations (NDD subgroup; *n* = 83). The autism diagnoses from these two measures had an agreement of 0.879 (*p* < 0.001) when evaluated using Cohen’s Kappa coefficient (Fleiss et al., 2013); this indicated that there was a substantially high agreement on the diagnosis of autism. The participants had a median age of 10 years, with the autism group 10 years, the NDD group 14 years, and the typically developing group 9 years. Please see Table 1 for more details on the participants. 53% (*n* = 41) of the children in the autism group and 9% (*n* = 8) in the NDD group were non-speaking. We did not administer cognitive reasoning tests such as the Ravens Progressive Matrices, which have been used in this setting before (Kitsao-Wekulo et al., 2013). As such, the cognitive functioning of the participants is not available. Currently, and at the time of data collection (2012–2013), the identification of neurodevelopmental disorders was not well developed, with limited healthcare and community-based facilities focusing on mental health and few psychiatrists and psychologists in Kilifi County (Bitta et al., 2017).

**Table 1:** Distribution of participant characteristics according to diagnostic groups

Participant characteristics	Autism	NDD	TD	Overall Sample
	(*n* = 78)	(*n* = 89)	(*n* = 101)	(*n* = 268)
Age in years:	10	14	9	10
Median (Q1, Q3)	(8, 13)	(11, 16)	(7, 11)	(8, 14)
Age groups				
Younger primary/special school aged (4–8 yrs)	26 (33.3%)	8 (9.0%)	34 (33.7%)	68 (25.6%)
Older primary/special school aged (9–13 yrs)	33 (42.3%)	36 (46.2%)	52 (51.5%)	121 (45.5%)
Adolescents (14–19 yrs)	15 (19.2%)	43 (48.3%)	15 (14.9%)	73 (27.4%)
Missing	4 (5.1%)	2 (2.2%)	0 (0.0%)	6 (1.5%)
Child sex				
Female	25 (32.1%)	37 (41.6%)	42 (41.6%)	104 (38.8%)
Male	53 (67.9%)	52 (58.4%)	59 (58.4%)	164 (61.2%)
Verbal (at least phrase speech) (SCQ Item 1)	37 (47.4%)	81 (91.0%)	101 (100.0%)	219 (81.7%)
Mother’s age in years (Median (Q1, Q3)	37 (32, 42)	37 (30, 41)	34 (29, 40)	36 (30, 42)
Father’s age in years (Median (Q1, Q3)	43 (37, 50)	45 (39, 53)	42 (36, 52)	44.5 (38, 52)
Maternal education				
Never attended	7 (7.1%)	38 (42.7%)	60 (24.1%)	105 (38.6%)
Primary	28 (42.9%)	34 (38.2%)	35 (14.1%)	97 (35.7%)
Secondary	23 (27.4%)	7 (7.9%)	3 (1.2%)	33 (12.1%)
Tertiary	0 (0%)	0 (0.0%)	0 (0.0%)	0 (0%)
Missing/Can’t recall	12 (14.3%)	10 (11.2%)	15 (5.2%)	37 (13.6%)
Paternal education				
Never attended	2 (2.4%)	14 (15.7%)	13 (5.9%)	29 (10.1%)
Primary	20 (23.8%)	42 (47.2%)	61 (27.7%)	123 (43.0%)
Secondary	24 (28.6%)	10 (11.2%)	13 (5.9%)	47 (16.4%)
Tertiary	12 (14.3%)	1 (1.1%)	3 (1.4%)	16 (5.6%)
Missing/Can’t recall	26 (31.0%)	22 (24.7%)	23 (8.0%)	71 (24.8%)

*NDD* neurodevelopmental disability, *TD* typically developing

### Measures

A socio-demographic questionnaire was designed by the study team and was used to collect information on ethnicity, language, and educational attainment.

The ADOS 2 was carried out by a special needs education specialist (JG), who received clinical training in administering and interpreting the ADOS 2. Opportunities for social interaction and communication were then observed in this standardised context. The ADOS 2 implemented in this study consists of four modules based on age and expressive language level. The ADOS 2 was performed on a randomly selected subset of the sample (*N* = 101) and coded (0 for non-autism and 1 for autism) by one of the co-authors (J.G.) in consultation with a developmental psychologist (AA) and a paediatric neurologist (CN). 83 of the 101 ADOS administrations were videotaped and assessed using the DSM-IV-TR criteria by a developmental psychologist (PK) and a developmental clinician (MK).

The lifetime version of the SCQ was administered in this study (Appendix 1). It is a brief 40-item Yes/No questionnaire that helps to evaluate communication skills and social functioning and is suggested for use in children above 4 years of age who may have autism (Berument et al., 1999). It is administered to a parent or other primary caregiver and takes less than 15 min. The instrument can be used for individuals above 4 years of age (chronological age) or at least 2 years (developmental age). It is available in two forms, lifetime and current; the lifetime form focuses on the child’s entire developmental history, while the current form is completed regarding the individual’s behaviour during the last three months, providing a total score that’s interpreted with specific cut-off points (Rutter et al., 2003). The presence of atypical behaviour is scored as a yes (coded 1) and the absence as no (coded 0). Non-verbal individuals have a lower total score, as the first seven items specific to language would be un-scorable (Rutter et al., 2003). In addition to the Total score, the SCQ can provide sub-scores for the ADI-R domains of Reciprocal Interaction (15 items), Communication (13 items), and Restricted, Repetitive and Stereotyped Patterns of Behaviour (8 items). Three additional items do not fall in these three domains when evaluated in the original validation by Berument et al. (2009) (item 1: level of speech, item 17: self-injury and item 38: attention to voice) (Rutter et al., 2003) and were therefore omitted from domain-wise analysis and factor analysis performed in this study. Although formal scoring of these sub-domains is not supported in the SCQ Auto Score materials, the manual fully supports researchers wanting to investigate these sub-domains.

While the ADOS is a one-time assessment of the child’s observed behaviour during administration, the SCQ-Lifetime queries the complete developmental history of the child and for the respondent to mention whether the behaviours have ever been present, with a specific focus on symptoms between 4 and 5 years of age. There are relatively few studies mapping out the phenotypic trajectories of autism across the years (Baghdadli et al., 2012; Georgiades et al., 2022; Gillespie-Lynch et al., 2012; Lord et al., 2015); however, most of these studies concede that autistic individuals show autism symptoms across the lifespan. One follow-up study found that there have been symptom changes between ages 2 and 15 that resulted in an improving class (Gotham et al., 2009). Longitudinal follow-up studies have also reported similar percentages of individuals who have reduced autism symptomatology as they age (Baghdadli et al., 2012; Billstedt et al., 2005; Gotham et al., 2009; Howlin et al., 2004). A recent longitudinal study by Elias and Lord (2022) highlighted that the majority of children who received an autism diagnosis in childhood continued to meet autism criteria even in adulthood, with 19% of participants with high cognitive ability (as evidenced by high IQ scores) no longer meeting autism diagnostic criteria. This same study notes gradual shifts in the social communication and repetitive behaviours domain (Elias & Lord, 2022). With these considerations in mind, comparisons between the ADOS-2 and the SCQ-Lifetime in this study are justifiable, particularly as some autistic individuals also had a co-occurring diagnosis of intellectual disability.

### Procedures

Extensive community engagement efforts were carried out with teachers, school administrators and parents of children in schools. Eligible parents and children were recruited from mainstream schools, special needs units and special schools. Typically developing children and children with a presumptive diagnosis of a neurodevelopmental condition (autism, severe learning disabilities and intellectual disability) from the Educational Assessment Resource Centre (EARC) were identified in the special schools.

A trained fieldworker shared information about the study and sought written consent to participate. They also interviewed parents/guardians and collected demographic and socio-economic data. An assessor (KR) trained by a developmental psychologist (AA) administered the SCQ to parents and guardians. The assessors were blind to the diagnostic status as much as possible, however, the certainty of the blinding procedures are varied as many caregivers and parents experienced the assessment as an opportunity to discuss their experiences caring for a non-typically developing child, as such assessors had an inkling in some instances of the case–control status, but not necessarily the exact diagnosis. The SCQ and the socio-demographic tools were translated into the local language, Kiswahili, through a standardised forward and back translation process as in previous studies. A panel/team involved in the translations included a developmental psychologist and trained professionals (linguists and research assistants) who were fluent in English and Kiswahili and familiar with the local culture.

### Statistical Analysis

Data was entered into MySQL and analysed using R statistical software (version 3.6.3) (R Development Core Team, 2020: https://www.r-project.org/).

#### Between-Group Comparisons

We compared the proportion of scores and the differences in total and sub-scale scores among the autism group and the typically developing group, the autism group and the NDD group using chi-squared tests and the Wilcoxon rank sum test with normally and non-normally distributed scores. Analysis of variance (ANOVA) was used to examine the relationship between the total score and subscale scores and the diagnosis groups. We conducted post-hoc analysis to also evaluate the relationship between the total and subscale scores with child age and non-verbal status. Child age was categorised as young school-aged/special school aged children (ages 4–8 years), older school aged/special school aged children (9–13 years) and older adolescents (14–19 years).

#### Factor Structure

We first carried out an exploratory factor analysis (EFA) to describe and summarise the SCQ items into a smaller number of latent factors. We used four methods to determine the number of factors to retain and rotate in EFA: (1) eigenvalues greater than 1.0 (Kaiser, 1960), (2) examining the scree plot (Cattell, 1966), (3) parallel analysis (PA) (Horn, 1965) and (4) interpretability, with the final deference to parallel analysis as the oft-recommended method of evaluating the dimensionality of a measure (Goretzko et al., 2021). Parallel analysis was carried out in R using the package *paran,* and we ran 5000 iterations.

There are also well-researched theories on the latent factors contributing to autism symptomatology; this includes the DSM-5 and DSM-IV. To assess the 3-factor DSM-IV (reciprocal social interaction, communication, and restricted repetitive behaviour) and 2-factor DSM-5 (social interaction and communication and restricted repetitive behaviour) models of the SCQ, we used confirmatory factor analysis (CFA) with maximum likelihood estimation. Table 2 describes which items were included in each of these factor models. Model fit was considered acceptable if the root mean squared error of approximation (RMSEA) was < 0.06 and if the Tucker–Lewis index (TLI) and comparative fit index (CFI) were both > 0.9 (Browne & Cudeck, 1992; Yu, 2002). Non-salient items (items with factor loadings < 0.30) were excluded to evaluate improvement in model fit. The packages *lavaan* (Revelle, 2019) and *semPlot* (Epskampe & Stuber, 2017) were used for factor analysis.

**Table 2:** Item endorsement frequencies according to diagnostic groups

SCQ subscale	SCQ Items	Autism	NDD	TD	Autism vs NDD	Autism vs TD
		(*n* = 78)	(*n* = 89)	(*n* = 101)	(*p*-value)	(*p*-value)
	1. At least phrase speech (If no, skip to question 8)	37 (47.4%)	81 (91.0%)	101 (100%)	< 0.001	< 0.001
Comm	2. Conversation*	18 (23.1%)	11 (12.4%)	0 (0.0%)	< 0.001	< 0.001
Comm	3. Stereotyped utterances*	20 (25.6%)	22 (24.7%)	4 (4.0%)	< 0.001	< 0.001
Comm	4. Inappropriate questions*	9 (11.5%)	15 (16.9%)	0 (0.0%)	< 0.001	< 0.001
Comm	5. Pronoun reversal*	15 (19.2%)	29 (32.6%)	5 (5.0%)	0.001	< 0.001
Comm	6. Neologisms*	13 (16.7%)	11 (12.4%)	2 (2.0%)	< 0.001	< 0.001
RRBI	7. Verbal rituals*	21 (26.9%)	29 (32.6%)	0 (0.0%)	< 0.001	< 0.001
RRBI	8. Compulsions and rituals	48 (61.5%)	16 (18.0%)	1 (1.0%)	< 0.001	< 0.001
Soc_Int	9. Inappropriate facial expressions	6 (7.7%)	9 (10.1%)	0 (0.0%)	0.784	0.006
Soc_Int	10. Use of other’s body	35 (44.9%)	2 (2.3%)	0 (0.0%)	< 0.001	< 0.001
RRBI	11. Unusual preoccupations	52 (66.7%)	46 (51.7%)	19 (18.8%)	0.071	< 0.001
RRBI	12. Repetitive use of objects	37 (47.4%)	20 (22.5%)	6 (5.9%)	0.001	< 0.001
RRBI	13. Circumscribed interests	45 (57.7%)	45 (50.6%)	36 (35.6%)	0.357	0.005
RRBI	14. Unusual sensory interests	49 (62.8%)	26 (29.2%)	5 (5.0%)	< 0.001	< 0.001
RRBI	15. Hand and finger mannerisms	20 (25.6%)	12 (13.5%)	0 (0.0%)	0.073	< 0.001
RRBI	16. Complex body mannerisms	32 (41.0%)	4 (4.5%)	0 (0.0%)	< 0.001	< 0.001
	17. Self-injury	33 (42.3%)	3 (3.4%)	0 (0.0%)	< 0.001	< 0.001
RRBI	18. Unusual attachment to objects	30 (38.5%)	29 (32.6%)	3 (3.0%)	0.528	< 0.001
Soc_Int	19. Friends	52 (66.7%)	33 (37.1%)	30 (29.7%)	< 0.001	< 0.001
Comm	20. Social chat	68 (87.2%)	58 (65.2%)	57 (56.4%)	0.002	< 0.001
Comm	21. Imitation	42 (53.8%)	33 (37.1%)	4 (4.0%)	0.044	< 0.001
Comm	22. Pointing to express interest	46 (59.0%)	30 (33.7%)	11 (10.9%)	0.002	< 0.001
Comm	23. Gestures	38 (48.7%)	20 (22.5%)	1 (1.0%)	< 0.001	< 0.001
Comm	24. Nodding to say yes	48 (61.5%)	13 (14.6%)	0 (0.0%)	< 0.001	< 0.001
Comm	25. Head shaking means no	51 (65.4%)	15 (16.9%)	0 (0.0%)	< 0.001	< 0.001
Soc_Int	26. Eye gaze	35 (44.9%)	23 (25.8%)	2 (2.0%)	0.016	< 0.001
Soc_Int	27. Social smiling	20 (25.6%)	6 (6.8%)	2 (2.0%)	0.002	< 0.001
Soc_Int	28. Showing and directing attention	41 (52.6%)	28 (31.5%)	3 (3.0%)	0.009	< 0.001
Soc_Int	29. Offering to share	50 (64.1%)	24 (27.0%)	8 (7.9%)	< 0.001	< 0.001
Soc_Int	30. Seeking to share enjoyment	45 (57.7%)	19 (21.3%)	6 (5.9%)	< 0.001	< 0.001
Soc_Int	31. Offering comfort	54 (69.2%)	46 (51.7%)	44 (43.6%)	0.032	< 0.001
Soc_Int	32. Quality of social overtures	25 (32.1%)	9 (10.1%)	1 (1.0%)	0.001	< 0.001
Soc_Int	33. Range official expressions	33 (42.3%)	18 (20.2%)	1 (1.0%)	0.003	< 0.001
Soc_Int	34. Imitative social play	51 (65.4%)	25 (28.1%)	3 (3.0%)	< 0.001	< 0.001
Soc_Int	35. Imaginative play	62 (79.5%)	42 (47.2%)	33 (68.8%)	< 0.001	< 0.001
Soc_Int	36. Interest in children	46 (59.0%)	23 (25.8%)	9 (8.9%)	< 0.001	< 0.001
Soc_Int	37. Response to other children	43 (55.1%)	17 (19.1%)	6 (5.9%)	< 0.001	< 0.001
	38. Attention to voice	32 (41.0%)	13 (14.6%)	6 (5.9%)	< 0.001	< 0.001
Soc_Int	39. Imaginative play with peers	65 (83.3%)	42 (47.2%)	8 (7.9%)	< 0.001	< 0.001
Soc_Int	40. Group play	60 (76.9%)	29 (32.6%)	3 (3.0%)	< 0.001	< 0.001

*NDD* neurodevelopmental disability, *TD* typically developing, *Comm* communication, *RRBI* restricted repetitive behaviours and interests, *Soc_Int* reciprocal social interaction; *p*-value from Pearson’s Chi-square test and Fisher’s exact test

^*^Items 2–7 are completed if a child has phrase speech, and the sample size in the autism group with phrase speech is 37 children

#### Reliability

We computed the internal consistency of the SCQ using the *psych* package (Revelle, 2019) and shared the Cronbach’s alpha (α) and McDonald’s omega (ω) of the overall items and per domain. The threshold for Cronbach’s α and Macdonald’s ω > 0.70 was considered satisfactory (Hair et al., 2010).

#### Diagnostic Accuracy

The receiver operating characteristic (ROC) curves (Hanley & McNeil, 1983) analysis is used to assess the validity of an instrument by plotting the true positive rate against the false positive rate. We computed the sensitivity, specificity and area under the ROC curve using the established cut-off (Berument et al., 1999) to determine how well the cut-off score of 15 distinguished between autistic individuals and the NDD and TD groups using the ADOS diagnoses and DSM-IV-TR clinical confirmations as the reference standard.

#### Item Response Theory

In addition to classical test theory, item response theory (IRT; Horn, 1965) helps evaluate the performance of tools at a more fine-grained resolution. We used an IRT approach to assess the relationship between the latent trait (autism) and item responses by evaluating the item/category response curves. Here, we make two assumptions: first, that the SCQ items measure the single latent trait of autism, and second, that autism may be a three-factor construct, with items in each factor contributing to the overall latent construct of autism. An essential component of IRT is the item response function—a mathematical function that relates the latent trait to the probability of endorsing an item. The item response function models the relationship between the participant trait level, item properties and the probability of endorsing the item. Item response function can then be converted to item characteristic curves (ICCs), which are a graphical representation of the participant’s trait level as a function of the probability of endorsing the item. Essential elements of the item response function include item difficulty and item discrimination. Difficulty reflects the proportion of endorsed items: the higher the difficulty parameter, the higher the trait level a participant needs to endorse the item. Discrimination captures the relationship between the item and the total score (latent trait). It describes how well the item distinguishes between people with different levels of the underlying trait.

Using the multidimensional item response theory (*mirt*) package (Chalmers, 2012) in R for dichotomous (two response) 2PL IRT models, we assessed the item functioning of the SCQ. A 2PL IRT model assumes that different items freely vary in their difficulty and discrimination. The RMSEA was used to test the model’s goodness of fit and to compute item parameters (Kline, 2015). We also plotted item characteristics and information curves to visualise how well each item in the SCQ contributes to scoring estimation precision: more informative items are expected to have broad coverage in the curves.

### Missing Data

The study sample consists of 268 participants. Thirty-nine participants were non-verbal, so the responses for items 2–7 (Table 2) were blank as these questions require verbal communication. For the psychometric analyses, such as factor analysis, we replaced the missing data with scores of 1 instead of 0 (out of 1) based on the premise that non-verbal autism may not be associated with later autism severity (Charman, 2005; Luyster et al., 2007). However, the findings on this assumption are mixed (Kjellmer et al., 2012). We chose to err on the side of caution as the assumption that non-verbal status is correlated with autism would lead to an over-endorsement of autism characteristics.

## Results

### Between-Group Comparisons

#### SCQ Item Endorsement Is Higher Amongst the Autism Group

Table 2 presents a detailed description of the SCQ item endorsement patterns across the study population and grouped by diagnosis. As seen from this table, the Autism group highly endorsed most of the items compared to the other diagnostic groups, with many of the items reaching statistical significance, apart from items 9, 11, 13, 15 and 18. Items 4, 5 and 7 were endorsed more for the NDD group (*p* ≤ 0.001).

#### The Autism Group Has Higher SCQ Total Scores

We used the F-statistic from analysis of variance (ANOVA)to compare mean SCQ scores obtained by those with autism, NDD and the TD groups. Overall, participants in the autism group had higher SCQ scores than other groups (Table 3) for the total mean scores (M = 18.9, SD = 7.88) and domain scores of reciprocal social interaction (M = 7.8), language and communication (M = 6.2), and repetitive and stereotyped patterns of behaviours (M = 3.9). However, when we further investigated the relationship between group differences and the total and sub-scale scores while adding child age and non-verbal status as covariates, we see that autistic children do have higher total and sub-scale scores compared to NDD and typically developing participants; child age was not a statistically significant predictor of the total score (*p* = 0.542). However, non-verbal status was statistically significant (*p* < 0.001) with a model coefficient of 12.07, suggesting that non-verbal children had, on average, a lower total score of 12.07 compared to verbal children. The adjusted R-squared value of the model (0.430) indicates that approximately 43% of the variance in the total score is explained by diagnostic status and non-verbal status. For the sub-scale scores, we see a similar pattern, with child age not being a significant predictor and non-verbal status being a significant predictor alongside diagnostic status, explaining 46% of the variance in the social reciprocity sub-scale, 30% in the communication sub-scale and 26% of the variance in the repetitive behaviour sub-scale (Supplementary Table 1). Males also had higher overall scores than females (Table 4) and higher scores in the language and communication domain. Males and females had identical mean scores in the repetitive and stereotyped patterns of behaviours domain.

**Table 3:** The autism group has higher SCQ total scores and domain scores in comparison to the NDD and TD groups

SCQ Scores	Overall sample	Autism (*n* = 78)	NDD (*n* = 89)	TD (*n* = 101)	Autism vs NDD vs TD *p*-value	Items
Total score: mean (SD)	10.0 (8.53)	18.9 (7.88)	10.1 (6.0)	2.9 (1.65)	F = 43.35, *p* = < 0.001	39
Reciprocal social interaction domain: mean (SD)	4.0 (3.89)	7.8 (3.94)	3.7 (3.00)	1.2 (1.07)	F = 24.23, *p* = < 0.001	15
Language and communication domain: mean (SD)	3.3 (2.98)	6.2 (2.78)	3.6 (2.32)	0.9 (0.84)	F = 53.22, *p* = < 0.001	13
Repetitive and stereotyped patterns of behaviours domain: mean (SD)	2.1 (2.07)	3.9 (2.00)	2.2 (1.86)	0.7 (0.85)	F = 33.84, *p* = < 0.001	8

*NDD* neurodevelopmental disability, *TD* typically developing, *F* = f-statistic, *p*-value from analysis of variance (ANOVA)

**Table 4:** SCQ Total Scores and domain scores for males and females

SCQ	Overall sample Male (*n* = 164)	Overall sample Female (*n* = 104)	Male vs Female p-value	Autism Male (*n* = 53)	Autism Female (*n* = 25)	NDD Male (*n* = 52)	NDD Female (*n* = 37)	TD Male (*n* = 59)	TD Female (*n* = 42)
Total score: mean (SD)	10.6 (8.78)	8.9 (8.05)	0.139	19.6 (7.43)	17.5 (8.76)	10.4 (5.68)	9.6 (6.41)	2.8 (1.60)	3.2 (1.72)
Reciprocal social interaction domain: mean (SD)	4.2 (4.13)	3.6 (3.44)	0.694	8.3 (3.80)	6.8 (4.10)	3.7 (2.93)	3.6 (3.13)	0.9 (0.98)	1.62 (1.08)
Language and communication domain: mean (SD)	3.6 (2.98)	2.9 (2.96)	0.042*	6.4 (2.45)	5.7 (3.40)	3.9 (2.21)	3.4 (2.47)	0.9 (0.82)	0.9 (0.86)
Repetitive and stereotyped patterns of behaviours domain: mean (SD)	2.2 (1.98)	2.2 (1.98)	0.085	3.9 (1.78)	4.0 (2.43)	2.3 (1.83)	2.2 (1.92)	0.8 (0.84)	0.5 (0.86)

*NDD* neurodevelopmental disability, *TD* typically developing, *p*-value from independent *t*-test

*Significant *p*-value

#### Older Children Have Higher SCQ Total Scores

Children with NDD were significantly older in age than autistic and typically developing children (*p* < 0.001). Adolescents also had higher total scores (M = 11.6, SD = 8.43) compared to younger and older primary school-aged children, as well as scores in the reciprocal social interaction (M = 4.7, SD = 3.92) and language and communication domain (M = 4.1, SD = 3.11). (Table 5)

**Table 5:** SCQ Total scores and domain scores according to age group

SCQ	Younger primary/special school aged (4–8)	Older primary/special school aged (9–13)	Adolescents (14–19)	Younger primary vs older primary vs adolescents *p*-value
Total score: mean (SD)	10.0 (9.36)	8.6 (7.96)	11.6 (8.43)	F = 2.84, *p* = 0.038
Reciprocal social interaction domain: mean (SD)	3.9 (4.24)	3.4 (3.57)	4.7 (3.92)	F = 3.09, *p* = 0.028
Language and communication domain: mean (SD)	3.0 (2.98)	3.0 (2.85)	4.1 (3.11)	F = 3.03, *p* = 0.030
Repetitive and stereotyped patterns of behaviours domain: mean (SD)	2.4 (2.42)	1.9 (1.87)	2.2 (2.06)	F = 1.49, *p* = 0.217

*F* f-statistic, *p*-value from analysis of variance (ANOVA)

### Parental Characteristics

There was no statistical difference in maternal and paternal ages among the three groups, as we can see in Table 1 [Maternal age (median, Q1, Q3) Autism—37 (32, 42), NDD—37 (30, 41) and TD—34 (29, 40); Paternal age (median, Q1, Q3) Autism—43 (37, 50), NDD—45 (39, 53) and TD—42 (36, 52). There were statistical differences in maternal and paternal education levels among the three groups (Table 1). It is important to note, however, that there is missing data related to paternal age and education level (~ 24% missingness). Maternal education was found to be significantly associated with the SCQ total scores.

## Factorial Structure of the SCQ

We first assessed the sampling adequacy of the SCQ data using the Kaiser–Meyer–Olkin (KMO) value and Bartlett’s Test of Sphericity. The KMO value was 0.93, which is greater than the > 0.50 threshold, and Bartlett’s test was statistically significant (*p* < 0.001), meaning we could therefore proceed with factor analysis.

### Exploratory Factor Analysis Reveals a 4-Factor Model for the Study Cohort

We first conducted an EFA to examine the underlying structure of the SCQ, as its dimensionality in our setting has not been researched in depth. We used principal axis factoring with oblique (oblimin) rotation. The first EFA generated six factors with eigenvalues greater than 1. Parallel analysis showed that four factors could be appropriately retained (see Supplementary Figs. 1 and 2; Supplementary Table 2). These exploratory analysis results can likely be interpretable as these four factors: factor 1 social communication and reciprocity; factor 2—unusual communication and mannerisms; factor 3—unusual non-verbal communication; and factor 4—restricted, repetitive behaviours and interests.

### Confirmatory Factor Analysis Supports a Three-Factor Model

We conducted a CFA to verify the two theoretical models of autism: the three-factor DSM-IV model of social reciprocity, communication and stereotyped behaviour and unusual interests and the two-factor DSM-5 model of social reciprocity and communication and stereotyped behaviour and unusual interests. We also assessed the tool developers’ four-factor model fit of the SCQ (Berument et al., 1999), social reciprocity, communication, abnormal language and stereotyped behaviour and the four-factor EFA model. Please see Supplementary Table 3 for a summary of which items were included in the models described below.

#### DSM-IV 3-Factor Model

The fit indices for the 3-factor DSM-IV model were excellent (RMSEA = 0.030, TLI = 0.993, CFI = 0.992). To further evaluate the factor structure, we omitted items with factor loadings below the 0.30 cut-off (non-salient items) (Browne & Cudeck, 1992) from the model and evaluated any change in the model fit. In the social reciprocity factor, we omitted 1/15 item (question 9-Inappropriate facial expressions); none in the communications factor, and in the stereotyped behaviour and unusual interests, we omitted 1/8 item (question 13-Circumscribed interests). After the omission of these items, the model improves with excellent fit indices (RMSEA = 0.028, TLI = 0.994, CFI = 0.994). We share detailed item factor loadings for the entire model and the revised model with omitted items in Supplementary Table 3.

#### DSM-5 2-Factor Model

The fit indices for the 2-factor DSM-5 model were excellent (RMSEA = 0.020, TLI = 0.997, CFI = 0.997). Only item 9 (inappropriate facial expressions) and item 13 (circumscribed interests) had a factor loading below the 0.30 cut-off (non-salient items). We share detailed item factor loadings for the whole model in Supplementary Table 4.

#### Berument et al., 4 Factor Model

The 4-factor Berument et al. model’s fit indices were also excellent (RMSEA = 0.012, TLI = 0.999, CFI = 0.999). Item 9 (Inappropriate facial expressions) in the restricted repetitive behaviour and interests factor had a factor loading below the 0.30 threshold. We share detailed item factor loadings for the full model in Supplementary Table 5. We did not rerun a revised model because there was only one non-salient item.

#### 4-Factor Kilifi SCQ Study

The fit indices for the 4-factor Kilifi model described in the EFA section above were excellent (RMSEA = 0.010, TLI = 0.99, CFI = 0.099). This model is different from the 4-factor model described above by Berument et al. in the composition of items in the four factors and interpretability of the factors. The Berument et al. model has factors that can be interpreted as social reciprocity, communication, abnormal language stereotyped behaviour and unusual interests with the items loading into them, as seen in Supplementary Table 5. In the Kilifi model, we see four factors that could be interpreted as social communication and reciprocity, unusual communication and mannerisms, non-verbal communication and restricted repetitive behaviour and interests. We share detailed item factor loadings for the full model and the revised model with omitted items in Supplementary Table 6. Only item 9 in the non-verbal communication factor had a factor loading below the 0.30 threshold. Just as above, there was only one non-salient item; therefore, we did not rerun a revised model.

With the results shared above, the 4-, 3-, and 2-factor models have adequate to excellent fit statistics, with the 4-factor models emerging as the best model fit indices (Supplementary Tables 3–6).

## Reliability of the SCQ

Internal consistency of the SCQ total scale in the overall group was excellent (Table 6). Good internal consistency coefficients were also observed for the Autism and NDD groups. The TD group, however, had poor internal consistency coefficients for all SCQ items [(α = 0.41 (0.25–0.57), Ω = 0.41 (0.25–0.57)].

**Table 6:** Above acceptable internal consistency of the SCQ for all items and three domains for diagnostic groups except the typically developing group

SCQ domains	Autism Cronbach’s α (95% CI)	NDD Cronbach’s α (95% CI)	TD Cronbach’s α (95% CI)	Overall sample Cronbach’s α (95% CI)	Autism McDonald’s Ω (95% CI)	NDD McDonald’s Ω (95% CI)	TD McDonald’s Ω (95% CI)	Overall sample McDonald’s Ω (95% CI)
All items	0.93 (0.90–0.95)	0.83 (0.78–0.88)	0.41 (0.25–0.57)	0.95 (0.94–0.96)	0.88 (0.02–0.91)	0.81 (0.75–0.76)	0.41 (0.25–0.57)	0.95 (0.94–0.96)
Reciprocal social interaction domain	0.85 (0.79–0.89)	0.77 (0.69–0.83)	0.39 (0.19–0.55)	0.89 (0.86–0.91)	0.81 (0.72–0.88)	0.76 (0.58–0.83)	0.12 (0.05–0.27)	0.88 (0.86–0.91)
Communication domain	0.74 (0.65–0.82)	0.72 (0.63–0.80)	0.22 (0.00–0.45)	0.91 (0.90–0.93)	0.67 (0.11–0.75)	0.50 (0.34–0.64)	0.22 (0.00–0.45)	0.80 (0.75–0.85)
Repetitive behaviours domain	0.70 (0.58–0.79)	0.68 (0.57–0.77)	0.39 (0.22–0.56)	0.80 (0.77–0.84)	0.63 (0.45–0.76)	0.67 (0.58–0.77)	0.39 (0.22–0.56)	0.79 (0.75–0.83)

In Table 6, we highlight good internal consistencies for the reciprocal social interaction and communication domains for the overall cohort and the Autism and NDD groups. We also note acceptable coefficients for the repetitive behaviour domain in the autism and NDD groups below. Again, however, we see poor internal consistency across all domains for the TD group.

Internal consistencies for all SCQ items in the male group [(α 0.93 (95% CI 0.92–0.95, Ω 0.94 (0.92–0.95)] and the female group were excellent [α 0.93 (95% CI 0.91–0.95), Ω 0.93 (0.90–0.94)]. Internal consistencies in the three domains were also good to excellent [reciprocal social interaction (overall = 0.88, male = 0.89, female = 0.84)], communication [overall = 0.84, male = 0.81, female = 0.83)], [repetitive behaviours = overall = 0.79, male = 0.72, female = 0.82)].

Overall, internal consistencies for the age groups were excellent [younger primary α 0.96 (95% CI 0.95–0.98), older primary α 0.95 (0.94–0.96), adolescents α 0.94 (0.91–0.96)]. Internal consistencies in the reciprocal social interaction and communication domain were also excellent, with lower Cronbach alphas in the repetitive behaviours’ domain, however they were still above the 0.70 threshold (Table 7). Cronbach alphas in each of the parental education groups were excellent [no formal schooling α 0.93 (95% CI 0.90–0.97), primary school (α 0.95 (95% CI (0.94–0.96) and secondary and beyond (α 0.95 (95% CI 0.94–0.97). For the social reciprocity, communication, and repetitive behaviours sub-scale, we see good to acceptable alphas (Table 7).

**Table 7:** Above acceptable internal consistency of the SCQ for all items in the age groups

SCQ domains	Younger primary/special school aged (4–8) Cronbach’s α (95% CI)	Older primary/special school aged (9–13) Cronbach’s α (95% CI)	Adolescents (14–19) Cronbach’s α (95% CI)	Overall sample Cronbach’s α (95% CI)	Parental education—no formal schooling Cronbach’s α (95% CI)	Parental education—primary schooling Cronbach’s α (95% CI)	Parental education—secondary schooling and beyond Cronbach’s α (95% CI)
All items	0.96 (0.95–0.98)	0.95 (0.94–0.96)	0.94 (0.91–0.96)	0.95 (0.94–0.96)	0.93 (0.90–0.97)	0.95 (0.94–0.96)	0.95 (0.94–0.97)
Reciprocal social interaction domain	0.91 (0.88–0.94)	0.88 (0.84–0.91)	0.87 (0.82–0.91)	0.89 (0.86–0.91)	0.78 (0.62–0.89)	0.89 (0.87–0.91)	0.86 (0.81–0.90)
Communication domain	0.94 (0.91–0.96)	0.92 (0.89–0.94)	0.89 (0.85–0.92)	0.91 (0.90–0.93)	0.89 (0.82–0.95)	0.90 (0.88–0.92)	0.92 (0.89–0.95)
Repetitive behaviours domain	0.88 (0.83–0.92)	0.77 (0.71–0.83)	0.76 (0.67–0.84)	0.80 (0.77–0.84)	0.76 (0.58–0.880	0.76 (0.71–0.81)	0.83 (0.77–0.88)

## Criterion Validity and Diagnostic Accuracy of the SCQ

The recommended cut-off of 15, as specified by the SCQ tool developers (Berument et al., 1999; Rutter et al., 2003), was reviewed against the scoring of the ADOS videos using the DSM-IV-TR criteria as the approximate reference standard to evaluate the diagnostic accuracy of the SCQ. The ADOS has high levels of diagnostic accuracy (Howes et al., 2018; Penner et al., 2018), particularly when used in combination with clinical judgement. The area under the ROC curve measures how well the overall SCQ total score can differentiate between a positive and a negative autism screen. The values range from 0 to 1, with values closer to 1 denoting excellent diagnostic accuracy. The recommended cut-off of 15 yielded an area under the curve score of 0.964, representing excellent diagnostic accuracy of the SCQ in differentiating autistic children and typically developing children. The cut-off point of 15 also yields a specificity of 100.0%, a sensitivity of 86.7%, a positive predictive value of 100% and a negative predictive value of 96.2%. The recommended cut-off point of 15 yielded an area under the curve score of 0.808, indicating a high possibility when differentiating between children with NDD and autistic children. We also see a specificity of 73.0%, sensitivity of 71.8%, positive predictive value of 70% and negative predictive value of 74.7%.

## Item Response Theory Approach of the SCQ

As we have seen in the analyses above, the SCQ is a multidimensional instrument; we, therefore, carried out a multidimensional IRT model to complement classical theory approaches to psychometric analysis. We also carried out multidimensional IRT using autism and NDD diagnosis as a further analysis grouping variable. Using the multidimensional item response theory for the dichotomous (two response) 2PL model, we found that the RMSEA for all items was below 0.06, indicating a good fit (Supplementary Table 7a). For the autism group, 20 of the items had a RMSEA below 0.06 (Supplementary Table 7b), while the NDD group had 26 items with a RMSEA below 0.06 (Supplementary Table 7c). We then generated IRT parameters (Supplementary Tables 8a) for each item. The values of the item discrimination slope ranged from 0.741 (item 19: Friends) to 4.530 (item 40: Group play), indicating moderate to strongly discriminative items and suggesting that all of the SCQ items discriminate respondents well along the latent trait of autism (Supplementary Table 8a). The item difficulty parameter estimates range from − 0.863 (item 20: Social chat) to 3.889 (item 9: Inappropriate facial expressions). In the autism group, item 15 (Hand and finger mannerisms) and item 12 (Repetitive use of objects) had the two lowest item discrimination slopes (0.146 and 0.260, respectively), while item 40 (Group play) and item 34 (Imitative social play) had the two highest item discrimination slopes at 30.729 and 8.259 respectively. Item 20 (Social chat) and 11 (Unusual preoccupations) had item difficulty parameters of − 1.690 and − 1.592, respectively; item 15 (Hand and finger mannerisms) and item 9 (Inappropriate facial expressions) had the two highest item difficulty parameters at 7.337, and 5.121 respectively. In the NDD group, the two lowest item discrimination parameters were − 0.179 (item 12: Repetitive use of objects) and 0.285 (item 17: Self-injurious behaviour), while the two highest item discrimination parameters were 2.547 (item 21: Imitation) and 2.448 (item 32: Quality of social overtures). The two lowest item difficulty parameters were − 6.969 (item 12: Repetitive use of objects) and − 0.557 (item 20: Social chat), while items 17 (Self-injurious behaviour) and 16 (Complex body mannerisms) had the highest difficulty parameters (11.893 and 8.392 respectively), more details on the parameters are in Supplementary Table 8c.

**Fig. 1:**
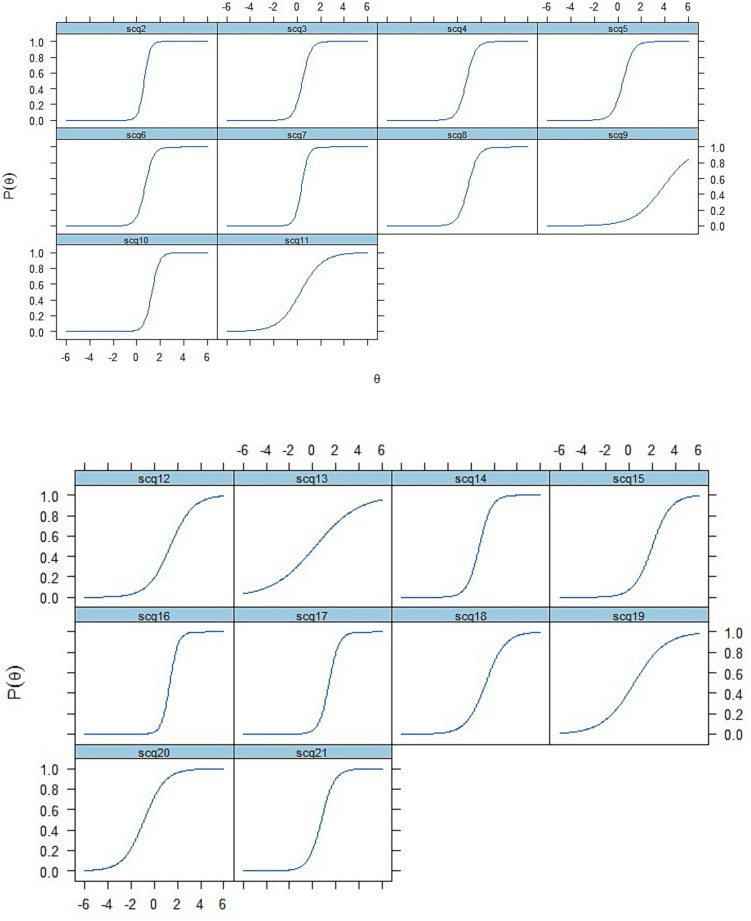

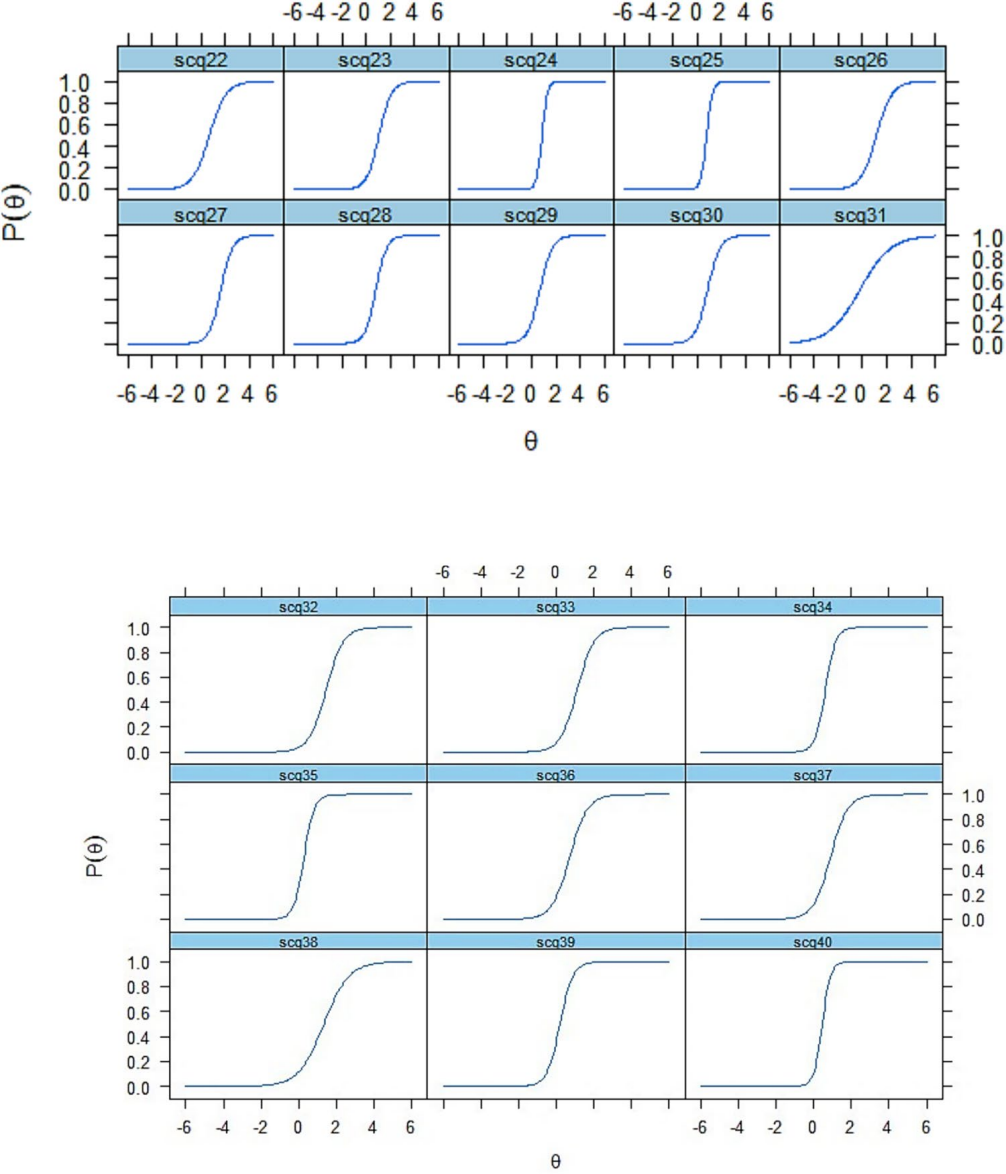
Item characteristic curves (ICCs) of the SCQ

We examined the probability of responding to specific options in an item’s response scale using item characteristic curves (ICCs) (Fig. 1). ICCs illustrate the slope of the latent trait, meaning that individuals with more of the latent trait, autism symptoms, have a higher chance of endorsing/passing the item.

We also evaluated how well each item contributed to the precision of score estimation by using item information curves (Supplementary Fig. 3), in which the steepness of the slope indicates how much information the item provides about the latent trait. Items 10 (Use of other’s body), 16 (Complex body mannerisms), 24 (Gestures) and 40 (Group play) have the steepest slopes, meaning that they provided the most information about the latent trait (autism).

## Discussion

This study evaluates the psychometric properties of the Kiswahili version of the SCQ in a diverse cohort of children and adolescents along the Kenyan Coast. There were significant differences in the SCQ scores between the autism, NDD, and typically developing groups. The recommended SCQ cut-off point of 15 yielded excellent sensitivity and specificity values. A 4-factor model emerged as the best fit for the underlying structure of the SCQ in our sample, although a 2-or 3-factor structure was also supported. Many of the SCQ items were discriminative and shared information about the latent construct of autism. These findings provide initial support for the use of the SCQ as an autism screening measure in children and adolescents along the Kenyan coast and similar settings.

The Autism group highly endorsed most of the SCQ items compared to the other diagnostic groups. Overall, participants in the autism group had significantly higher total and domain-specific scores than both controls and participants with other NDDs, demonstrating the specificity of this tool as a diagnostic screen to identify autism and discriminate it from other related neurodevelopmental conditions. This finding is consistent with other studies investigating the performance of the SCQ (Chandler et al., 2007; Corsello et al., 2007; Magyar et al., 2012; Zarokanellou et al., 2017), where higher scores are noted for individuals diagnosed with autism. A study in China by Gau et al. (2011) also found significant differences in total scores between males and females, a finding that we also observed where males also had higher overall and domain scores with significantly higher scores in the communication domain. We observed that non-verbal status affected the total and sub-scale scores. Limited communication and social skills have been found to be indicative of autism (Bryson et al., 2007; Toth et al., 2006). One study has looked at the internal consistency of the SCQ in verbal and non-speaking children have found high Cronbach’s alphas (0.94 and 0.89), respectively and good sensitivity and specificity in both groups with a cut-off of 12 (Marvin et al., 2017). Another study that evaluated lower cut-off points found a lower cut-off score of 12 when they included non-speaking children in the analysis (Karaminis & Stavrakaki, 2022). We were not able to explore different cutoff scores in the study; this could be an avenue for replication of these analyses with more participants to evaluate whether different cutoff points moderate the effects of verbal status on SCQ scores.

Our factor analytic structure differs somewhat from other findings, with exploratory factor analysis showing that a four-factor solution was most appropriate for the data. Magyar et al. (2012) and Snow and Lecavalier (2008) found that both EFA and CFA supported a 2-factor structure appropriate and consistent with the current DSM-5 conceptualisation of autism, social communication/interaction and restricted, repetitive patterns of behaviour, interests, or activities. We interpreted our four factors as: (i) social communication and reciprocity, (ii) unusual communication and mannerisms, (iii) non-verbal communication and (iv) restricted repetitive behaviour and interests. Our confirmatory factor analysis supports 2, 3 and 4-factor solutions, with each having excellent model fit statistics and the 4-factor solution emerging as the best fit. This finding is corroborated by the original validation study by Berument et al. (1999) and the Greek and Chinese version validations of the SCQ (Gau et al., 2011; Zarokanellou et al., 2017). In all the models evaluated, item 9, “Has her/his facial expression usually seemed inappropriate to the particular situation, as far as you could tell?” (Inappropriate facial expressions), in the reciprocal social communication domain, emerged as non-salient. It is also interesting to note that this item was endorsed slightly more in the NDD group (9) than in the autism group (6), although this comparison was not statistically significant. There is evidence that individuals with autism make fewer facial expressions and have difficulty making appropriate ones at the right time (Czapinski & Bryson, 2003). This may lead to their facial expressions being interpreted as ambiguous, odd or mechanical (Faso et al., 2015). The ability to make facial expressions, a term conceptualised as the visual appearance of facial expressions (Trevisan et al., 2018), represents the degree to which facial expressions appear consistent with neurotypical norms and convey the intended emotion. Using facial expressions in an accurate neurotypical fashion relies on an understanding of social contexts and the emotional and mental state of the other person (Kappas et al., 2013). There is also evidence that some individuals with autism have challenges recognising basic and complex emotions, with some cultural differences noted (Fridenson-Hayo et al., 2016). It is therefore plausible that this facial expressivity, as a concept, is also culturally sensitive and perhaps is differently evaluated in our culture compared to other settings. It is perhaps readily associated with neurodevelopmental differences rather than just autism differences. This may explain the non-saliency of the item as well as the endorsement pattern of this item in both the Autism and NDD groups.

Internal consistency of the SCQ total score in the overall group was excellent. Internal consistencies for all SCQ items in the Autism and NDD diagnostic categories were good as well as child age categories and parental education categories This is a similar finding as in the study by Marvin et al. (2017), who found Cronbach alphas of 0.94 for verbal children and 0.89 for non-verbal children. More studies have reported more modest internal reliability coefficients of around 0.80 as in the Chinese version of the SCQ (Gau et al., 2011), in the German version (Bölte et al., 2008) and the Turkish version (Avcil et al., 2015). Snow and Lecavalier (2008) also found good internal consistency (0.81). In these studies mentioned above, they only included participants at risk or with an autism diagnosis. In our study, we included typically developing children, and we noted poor internal consistency coefficients in this group. This seems reasonable, given that the SCQ is an autism screening tool and is not expected to index behaviours of typically developing children. We also observed low scores with the typically developing group (with an average score of 2.9); as such, this limited variability in scores may have led to lower internal consistency.

The recommended cut-off of 15 yielded an area under the curve score of 0.964, representing very good diagnostic accuracy of the SCQ. The cut-off point of 15 also yields a specificity of 100.0% and a sensitivity of 86.7%. The original validity study of the SCQ by Berument et al. (1999) found that the recommended cut-off of 15 yielded a sensitivity of 85% and a specificity of 75%. Magyar and colleagues found an AUC of 0.779. Another study by Bolte et al. found that the cut-off of 15 differentiated between autism and other conditions, with a sensitivity of 89% and a specificity of 91%. The SCQ also performed well with a sensitivity of 92% and sensitivity of 94% in a study by (Witwer & Lecavalier, 2007) and a sensitivity of 96.3% and a specificity of 98.7% for the Autism group versus the Non-Spectrum group in a study in Greece (Zarokanellou et al., 2017). This is again a consistent finding in the previous validation studies of the SCQ. We do, however, have only a subset 83/268 of the entire study sample with validated diagnoses for comparison; this may have contributed to the high specificity we have noted here.

The values of the discrimination slope indicated that most of the SCQ items discriminate respondents well along the latent trait that is autism. A study by Wei et al. (2015) also found that most of the items in the SCQ Lifetime had high discrimination properties. In the entire study sample and in the autism group, item 40 (Group play) was the most discriminating item, and item 5 (Pronoun reversal) and item 15 (Hand and finger mannerisms) was the least discriminating. The item difficulty parameters were also robust in the entire sample. Items 9 (Inappropriate facial expressions), 15 (Hand and finger mannerisms)), 24 (Gestures), and 40 (Group play) had the most information about the latent trait Item 12 (Repetitive use of objects) in the NDD group. In our study, items in the repetitive behaviours domain appear to have lower discrimination and difficulty parameters compared to communication social interaction and reciprocity domain. Repetitive behaviours are one of the core domains in autism diagnosis; they are varied and are present, to some extent, in typically developing children, autistic children and children with other developmental conditions (Scahill et al., 2015). Studies have put forward high co-occurring rates between autism and intellectual disability, ranging from 40–60%, as noted in a review by Buescher and colleagues (Buescher et al., 2014). It is useful to have measures that have robust discriminating autistic traits in autistic individuals with intellectual disability. With a number of the items having moderate to high item difficulty parameters, the SCQ has items that would be able to assess mild to more severe autistic traits. This lends support to the internal validity of the SCQ-Lifetime as an autism screening tool.

## Limitations of the Study and Future Consideration

This present study is one of the few evaluations of the psychometric properties of the SCQ in Africa. While there are a number of strengths in the study, it is important to interpret the findings of our study with some considerations in mind. We acknowledge that we have a relatively modest sample size, which means we could not explore the sensitivity and specificity of alternative cut-off scores of the SCQ in our setting. We also did not administer the ADOS 2 or videotape all ADOS 2 administrations in the entire study sample, so diagnostic comparisons were not available for all participants. We did not have scoring from different raters or repeated assessment information as such we could not discuss other forms of reliability such as inter-rater reliability or test–retest reliability. We also have a varied age group and an unequal number of participants in each age band. This means we could not explore the age-dependent performance of the SCQ, thereby not being able to add information on whether the SCQ performs better with older children vs. younger children in our setting, something other studies of the SCQ have explored in more detail. We also did not have information on the children’s cognitive functioning as such we were not able to comment on the differences in the SCQ scores due to cognitive functioning level. We also only had responses from caregivers. It might be useful to compare responses from additional information, such as teachers, which might be helpful in further evaluating the clinical utility of the SCQ. The SCQ was designed to be a self-rated questionnaire; however, with the relatively higher levels of non-literacy in our setting, the instrument was adapted to be administered to the caregivers via interview. While we used the ADOS and DSM-IV-TR clinical confirmation, we would like to highlight the absence of a formal ‘gold standard’ autism diagnostic tool in our setting. We also assumed that all non-verbal children did not endorse verbal items 2–7 in the SCQ when evaluating the item endorsement characteristics. This may contribute to the under-endorsement of autism traits; however, as mentioned, we chose to err on the side of caution.

## Conclusion

Our findings show excellent internal consistency properties and good discriminative properties of the SCQ-Lifetime with significant differences in scores for the autism group, the NDD group and the typically developing group. This suggests that the SCQ is a potentially clinically useful instrument in the screening of autism in a Kenyan context and further discrimination of autism from other NDDs. A four-factor model emerged during the exploratory analysis, and confirmatory factor analysis yielded excellent fit statistics for a two, three and four-factor model. The established cut-off of 15 was discriminative of children in the autism group with good sensitivity and specificity. In summary, the Kiswahili version of the SCQ-Lifetime shows good psychometric properties. It would be appropriate for use in school and community settings in Kilifi and other parts of Kenya as a screening questionnaire for autism. This scale is relatively quick and easy to administer. It would aid in bridging the gap between early identification and referral for care, which is urgently needed to improve efforts for early intervention that can improve lifelong trajectories of patient health and wellbeing.

## Supplementary Information

Below is the link to the electronic supplementary material.

Supplementary file1 (DOCX 769 KB)
