# Correction: Evaluation of the Psychometric Properties of the Social Communication Questionnaire in Rural Kenya

**DOI:** 10.1007/s10803-025-07169-0

**Published:** 2025-12-02

**Authors:** Patricia Kipkemoi, Jeanne E. Savage, Joseph Gona, Kenneth Rimba, Martha Kombe, Paul Mwangi, Collins Kipkoech, Danielle Posthuma, Charles R. J. C. Newton, Amina Abubakar

**Affiliations:** 1 Neuroscience Unit, KEMRI-Wellcome Trust Research Programme, 230-80108 Kilifi, Kenya; 2 Complex Trait Genetics Department, Center for Neurogenomics and Cognitive Research (CNCR), Vrije Universiteit Amsterdam, Amsterdam, Netherlands; 3 Department of Child and Adolescent Psychology and Psychiatry, Complex Trait Genetics, Amsterdam University Medical Centres, Vrije Universiteit Amsterdam, Amsterdam Neuroscience, Amsterdam, The Netherlands; 4 Department of Psychiatry, Warneford Hospital, University of Oxford, Warneford Ln, Oxford, OX3 7JX UK; 5 Department of Public Health, Pwani University, Kilifi, 195-80108, Kenya; 6 Institute for Human Development, Aga Khan University, Nairobi, 30270-00100 Kenya

**Correction to: Journal of Autism and Developmental Disorders (2024) 55:2919–2937**

10.1007/s10803-024-06380-9

In this article, the Appendix section contains a questionnaire which was printed without copyright permission has been removed.

The original article has been corrected.

