# Supplementary material for: Evaluation of the Psychometric Properties of the Social Communication Questionnaire in Rural Kenya
Source: J Autism Dev Disord. 2024 May 30;55(8):2919–37. doi: 10.1007/s10803-024-06380-9 (PMC7616653; doi:10.1007/s10803-024-06380-9)
Supplement: Supplementary file 1 — Supplementary file1 (DOCX 769 KB) [file 10803_2024_6380_MOESM1_ESM.docx]

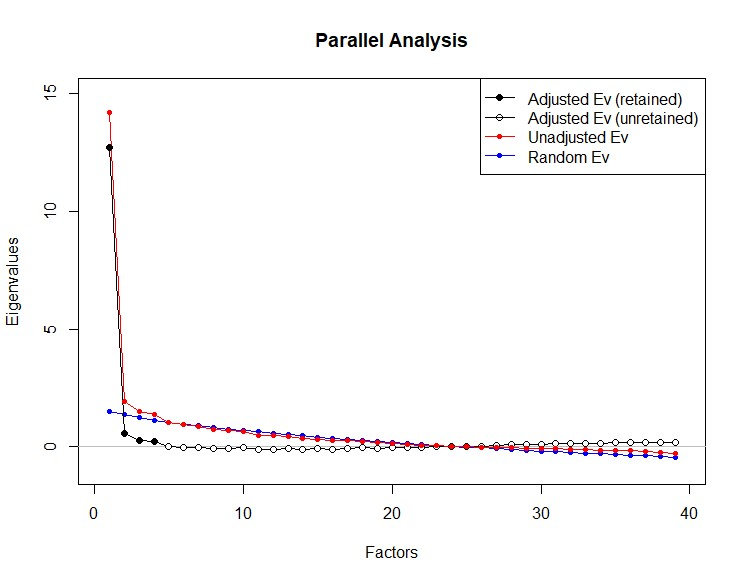


*Supplementary Figure 1. Parallel analysis for factors to retain for exploratory factor analysis*


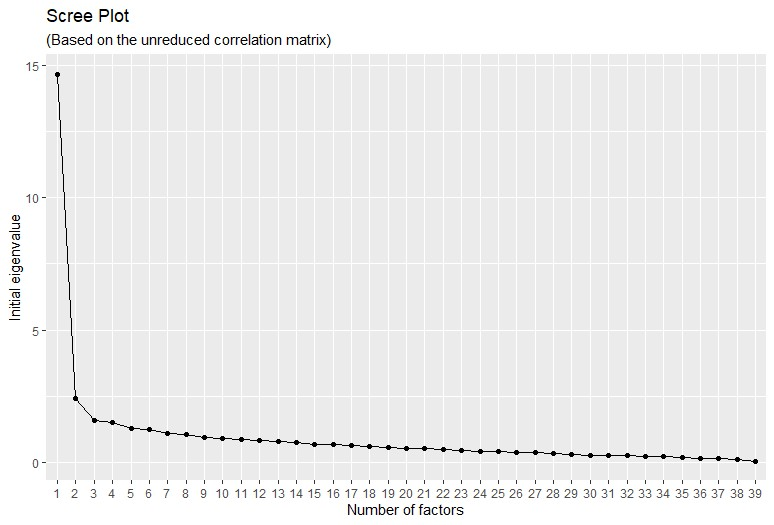


Supplementary Figure 2: Scree Plot for exploratory factor analysis shows 4 factors that can be retained


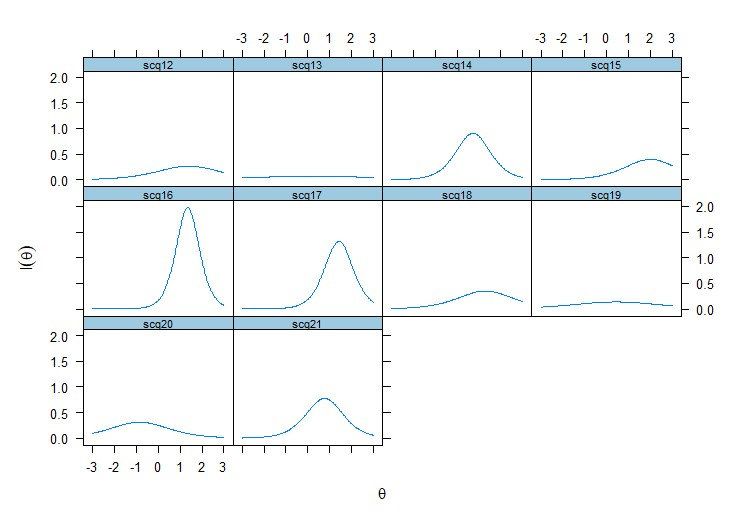

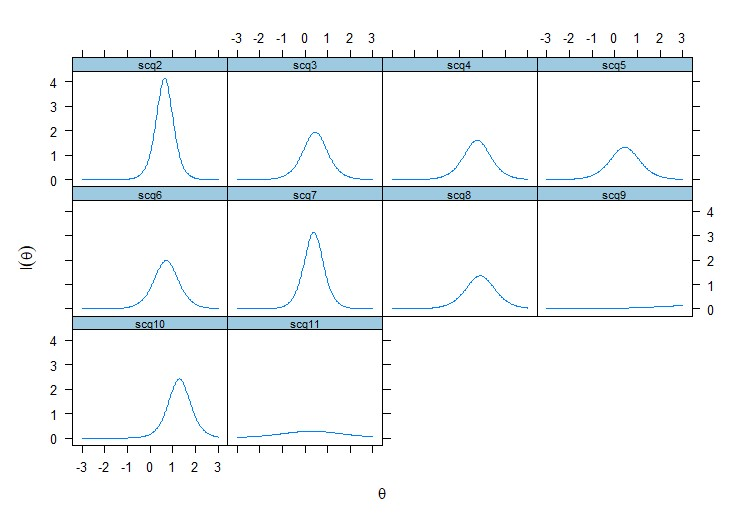


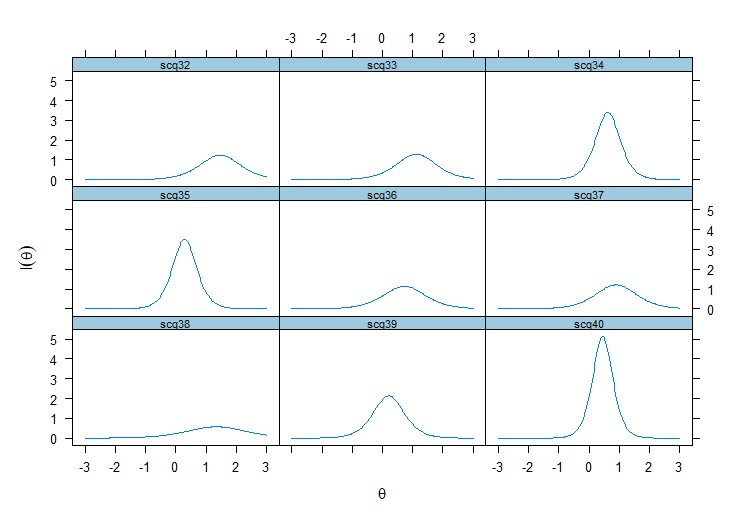

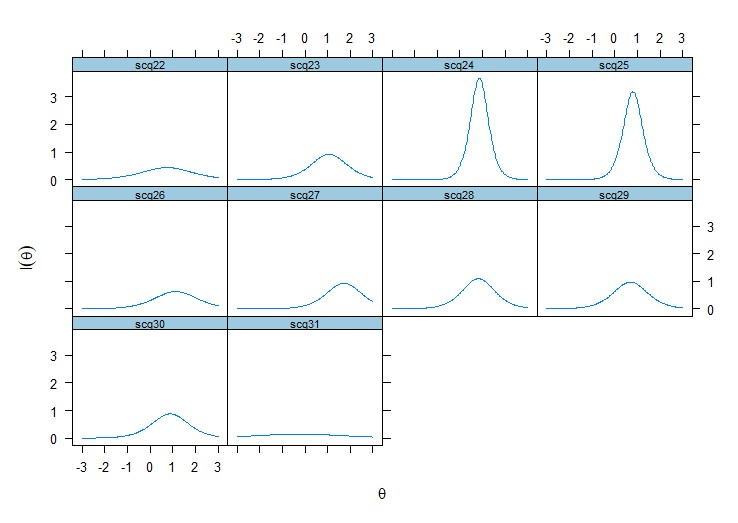


Supplementary Figure 3: Information Item Curves of the SCQ Items

| Social Reciprocity Subscale | | | | |
| --- | --- | --- | --- | --- |
| Predictor | Estimate | Standard Error | T-value | P-value |
| (Intercept) | 7.426 | 0.605 | 12.267 | < 0.001 |
| Diagnostic group | 0.854 | 0.228 | 3.744 | < 0.002 |
| Child age in years | 0.072 | 0.045 | 1.587 | 0.114 |
| Non-verbal status | 6.251 | 0.458 | -13.649 | < 0.001 |
| Communication Subscale | | | | |
| Predictor | Estimate | Standard Error | T-value | P-value |
| (Intercept) | 4.225 | 0.529 | 7.993 | < 0.001 |
| Diagnostic group | 1.246 | 0.199 | 6.252 | < 0.001 |
| Child age in years | 0.023 | 0.040 | 0.580 | 0.562 |
| Non-verbal status | -2.877 | 0.400 | -7.194 | < 0.001 |
| Repetitive Behaviour Subscale | | | | |
| Predictor | Estimate | Standard Error | T-value | P-value |
| (Intercept) | 3.390 | 0.379 | 8.945 | < 0.001 |
| Diagnostic group | 0.778 | 0.143 | 5.446 | < 0.001 |
| Child age in years | -0.032 | 0.028 | -1.114 | 0.266 |
| Non-verbal status | -2.034 | 0.287 | -7.094 | < 0.001 |

*Supplementary Table 1: Regression Model for age, non-verbal status and diagnostic group on SCQ sub-scale scores i. social reciprocity, ii. communication and repetitive behaviour*

| **Items** | **Factor 1 - Social communication and reciprocity** | **Factor 2 - Unusual communication and mannerisms** | **Factor 3 - Unusual non-verbal communication** | **Factor 4 - Restricted repetitive behaviour and interests** |
| --- | --- | --- | --- | --- |
| 2. Conversation | 0.280 | **0.430** | 0.070 | 0.220 |
| 3. Stereotyped utterances | 0.070 | **0.690** | 0.050 | 0.060 |
| 4. Inappropriate questions | 0.040 | **0.890** | -0.020 | -0.140 |
| 5. Pronoun reversal | -0.030 | **0.700** | 0.040 | 0.150 |
| 6. Neologisms | -0.020 | **0.900** | 0.010 | -0.020 |
| 7. Verbal rituals | -0.040 | **0.710** | 0.080 | 0.280 |
| 8. Compulsions and rituals | 0.320 | **0.340** | -0.070 | 0.190 |
| 9. Inappropriate facial expressions | -0.220 | 0.170 | **0.350** | 0.010 |
| 10. Use of other’s body | **0.550** | 0.260 | -0.240 | 0.110 |
| 11. Unusual preoccupations | -0.030 | 0.150 | -0.030 | **0.560** |
| 12. Repetitive use of objects | -0.010 | 0.250 | -0.140 | **0.430** |
| 13. Circumscribed interests | -0.120 | 0.050 | -0.010 | **0.540** |
| 14. Unusual sensory interests | 0.280 | 0.090 | 0.020 | **0.470** |
| 15. Hand and finger mannerisms | -0.050 | **0.360** | 0.080 | 0.100 |
| 16. Complex body mannerisms | **0.490** | 0.190 | -0.070 | 0.080 |
| 17. Self-injurious behaviour | **0.630** | 0.180 | -0.360 | 0.100 |
| 18. Unusual attachment to object | 0.070 | -0.090 | 0.140 | **0.580** |
| 19. Friends | **0.350** | -0.030 | -0.060 | 0.130 |
| 20. Social chat | 0.160 | 0.030 | 0.200 | 0.200 |
| 21. Imitation | 0.100 | 0.080 | **0.650** | 0.080 |
| 22. Pointing to express interest | **0.490** | -0.020 | 0.200 | -0.050 |
| 23. Gestures | 0.290 | 0.290 | 0.160 | -0.020 |
| 24. Nodding to say yes | **0.860** | -0.070 | 0.060 | 0.020 |
| 25. Head shaking to mean no | **0.870** | -0.050 | 0.060 | 0.000 |
| 26. Eye gaze | 0.170 | 0.290 | 0.160 | 0.040 |
| 27. Social smiling | **0.510** | -0.030 | 0.080 | -0.010 |
| 28. Showing and directing attention | 0.340 | 0.150 | **0.410** | -0.070 |
| 29. Offering to share | **0.450** | 0.070 | 0.100 | 0.190 |
| 30. Seeking to share enjoyment | **0.410** | 0.160 | 0.330 | -0.120 |
| 31. Offering comfort | 0.230 | 0.000 | 0.120 | 0.120 |
| 32. Quality of social overtures | 0.230 | 0.240 | **0.390** | -0.110 |
| 33. Range of facial expressions | 0.130 | 0.170 | **0.470** | 0.150 |
| 34. Imitative social play | **0.440** | 0.100 | 0.340 | 0.150 |
| 35. Imaginative play | **0.370** | 0.050 | 0.380 | 0.270 |
| 36. Interest in children | **0.500** | 0.220 | 0.160 | -0.120 |
| 37. Response to other children | **0.590** | 0.150 | 0.110 | -0.110 |
| 38. Attention to voice | **0.370** | 0.120 | 0.100 | 0.050 |
| 39. Imaginative play with peers | **0.420** | 0.150 | 0.200 | 0.170 |
| 40. Group play | **0.470** | 0.130 | 0.260 | 0.220 |

*Supplementary Table 2: Exploratory Factor Analysis of the SCQ*

Factor loadings for the 39 items in the SCQ; items highlighted in green indicate items with a factor loading threshold of above 0.300 recommended cut-off point.

| **Items** | **Factor 1 -Social reciprocity** | **Factor 2 - Communication** | **Factor 3 - Stereotyped behaviour and unusual interests** |
| --- | --- | --- | --- |
| 2. Conversation |  | **0.796** |  |
| 3. Stereotyped utterances |  | **0.732** |  |
| 4. Inappropriate questions |  | **0.682** |  |
| 5. Pronoun reversal |  | **0.687** |  |
| 6. Neologisms |  | **0.728** |  |
| 7. Verbal rituals |  |  | **0.877** |
| 8. Compulsions and rituals |  |  | **0.726** |
| 9. Inappropriate facial expressions | 0.136 |  |  |
| 10. Use of other’s body | **0.636** |  |  |
| 11. Unusual preoccupations |  |  | **0.466** |
| 12. Repetitive use of objects |  |  | **0.413** |
| 13. Circumscribed interests |  |  | 0.283 |
| 14. Unusual sensory interests |  |  | **0.687** |
| 15. Hand and finger mannerisms |  |  | **0.412** |
| 16. Complex body mannerisms |  |  | **0.656** |
| 19. Friends | **0.342** |  |  |
| 20. Social chat |  | **0.415** |  |
| 21. Imitation |  | **0.594** |  |
| 22. Pointing to express interest |  | **0.505** |  |
| 23. Gestures |  | **0.579** |  |
| 24. Nodding to say yes |  | **0.748** |  |
| 25. Head shaking to mean no |  | **0.751** |  |
| 26. Eye gaze | **0.525** |  |  |
| 27. Social smiling | **0.466** |  |  |
| 28. Showing and directing attention | **0.657** |  |  |
| 29. Offering to share | **0.649** |  |  |
| 30. Seeking to share enjoyment | **0.632** |  |  |
| 31. Offering comfort | **0.368** |  |  |
| 32. Quality of social overtures | **0.565** |  |  |
| 33. Range of facial expressions | **0.640** |  |  |
| 34. Imitative social play |  | **0.803** |  |
| 35. Imaginative play |  | **0.822** |  |
| 36. Interest in children | **0.677** |  |  |
| 37. Response to other children | **0.664** |  |  |
| 39. Imaginative play with peers | **0.746** |  |  |
| 40. Group play | **0.843** |  |  |

*Supplementary Table 3: Factor loadings for the DSM-IV- Three-factor confirmatory factor analysis models.*

Factor loadings for the 39 items in the SCQ; items highlighted in green indicate items with a factor loading threshold of above 0.300 recommended cut-off point and items in red are below this threshold.

| **Items** | **F1 -Social reciprocity and communication** | **F2 - Stereotyped behaviour and unusual interests** |
| --- | --- | --- |
| 2. Conversation | **0.803** |  |
| 3. Stereotyped utterances |  | **0.802** |
| 4. Inappropriate questions | **0.684** |  |
| 5. Pronoun reversal |  | **0.755** |
| 6. Neologisms |  | **0.801** |
| 7. Verbal rituals |  | **0.859** |
| 8. Compulsions and rituals |  | **0.712** |
| 9. Inappropriate facial expressions | **0.135** |  |
| 10. Use of other’s body | **0.629** |  |
| 11. Unusual preoccupations |  | **0.459** |
| 12. Repetitive use of objects |  | **0.409** |
| 13. Circumscribed interests |  | **0.278** |
| 14. Unusual sensory interests |  | **0.670** |
| 15. Hand and finger mannerisms |  | **0.405** |
| 16. Complex body mannerisms |  | **0.640** |
| 19. Friends | **0.339** |  |
| 20. Social chat | **0.418** |  |
| 21. Imitation | **0.605** |  |
| 22. Pointing to express interest | **0.515** |  |
| 23. Gestures | **0.585** |  |
| 24. Nodding to say yes | **0.761** |  |
| 25. Head shaking to mean no | **0.764** |  |
| 26. Eye gaze | **0.519** |  |
| 27. Social smiling | **0.461** |  |
| 28. Showing and directing attention | **0.650** |  |
| 29. Offering to share | **0.642** |  |
| 30. Seeking to share enjoyment | **0.625** |  |
| 31. Offering comfort | **0.364** |  |
| 32. Quality of social overtures | **0.560** |  |
| 33. Range of facial expressions | **0.634** |  |
| 34. Imitative social play | **0.797** |  |
| 35. Imaginative play | **0.792** |  |
| 36. Interest in children | **0.669** |  |
| 37. Response to other children | **0.657** |  |
| 39. Imaginative play with peers | **0.737** |  |
| 40. Group play | **0.833** |  |

*Supplementary Table 4: Factor loadings for the DSM-5 two-factor confirmatory factor analysis models*

Factor loadings for the 39 items in the SCQ; items highlighted in green indicate items with a factor loading threshold of above 0.300 recommended cut-off point and items in red are below this threshold.

| **Items** | **F1 -Social reciprocity** | **F2 - Communication** | **F3 - Abnormal language** | **F4 - Stereotyped behaviour and unusual interests** |
| --- | --- | --- | --- | --- |
| 2. Conversation |  | **0.815** |  |  |
| 3. Stereotyped utterances |  | **0.745** |  |  |
| 4. Inappropriate questions |  |  | **0.770** |  |
| 5. Pronoun reversal |  |  | **0.773** |  |
| 6. Neologisms |  |  | **0.820** |  |
| 7. Verbal rituals |  |  | **0.084** |  |
| 8. Compulsions and rituals |  |  | **0.535** |  |
| 9. Inappropriate facial expressions |  |  |  | 0.142 |
| 10. Use of other’s body |  | **0.640** |  |  |
| 11. Unusual preoccupations |  |  |  | **0.490** |
| 12. Repetitive use of objects |  |  |  | **0.432** |
| 13. Circumscribed interests |  |  |  | **0.301** |
| 14. Unusual sensory interests |  |  |  | **0.709** |
| 15. Hand and finger mannerisms |  |  |  | **0.423** |
| 16. Complex body mannerisms |  | **0.609** |  |  |
| 17. Self-injurious behaviour |  |  |  | **0.624** |
| 18. Unusual attachment to object | **0.429** |  |  |  |
| 19. Friends |  |  |  | **0.379** |
| 20. Social chat | **0.428** |  |  |  |
| 21. Imitation | **0.605** |  |  |  |
| 22. Pointing to express interest | **0.524** |  |  |  |
| 23. Gestures | **0.592** |  |  |  |
| 24. Nodding to say yes |  | **0.765** |  |  |
| 25. Head shaking to mean no |  | **0.767** |  |  |
| 26. Eye gaze | **0.526** |  |  |  |
| 27. Social smiling | **0.473** |  |  |  |
| 28. Showing and directing attention | **0.660** |  |  |  |
| 29. Offering to share | **0.655** |  |  |  |
| 30. Seeking to share enjoyment | **0.635** |  |  |  |
| 31. Offering comfort | **0.372** |  |  |  |
| 32. Quality of social overtures | **0.559** |  |  |  |
| 33. Range of facial expressions | **0.642** |  |  |  |
| 34. Imitative social play | **0.809** |  |  |  |
| 35. Imaginative play | **0.801** |  |  |  |
| 36. Interest in children | **0.681** |  |  |  |
| 37. Response to other children | **0.670** |  |  |  |
| 38. Attention to voice | **0.589** |  |  |  |
| 39. Imaginative play with peers | **0.749** |  |  |  |
| 40. Group play | **0.844** |  |  |  |

*Supplementary Table 5: Factor loadings for the four factors (from the original developers Berument et al.,) confirmatory factor analysis model*

Factor loadings for the 39 items in the SCQ; items highlighted in green indicate items with a factor loading threshold of above 0.300 recommended cut-off point and items in red are below this threshold.

| **Items** | **Factor 1 - Social communication and reciprocity** | **Factor 2 - Unusual communication and mannerisms** | **Factor 3 - Non-verbal communication** | **Factor 4 - Restricted repetitive behaviour and interests** |
| --- | --- | --- | --- | --- |
| 2. Conversation |  | **0.855** |  |  |
| 3. Stereotyped utterances |  | **0.788** |  |  |
| 4. Inappropriate questions |  | **0.738** |  |  |
| 5. Pronoun reversal |  | **0.741** |  |  |
| 6. Neologisms |  | **0.785** |  |  |
| 7. Verbal rituals |  | **0.844** |  |  |
| 8. Compulsions and rituals |  | **0.703** |  |  |
| 9. Inappropriate facial expressions |  |  | 0.147 |  |
| 10. Use of other’s body | **0.637** |  |  |  |
| 11. Unusual preoccupations |  |  |  | **0.590** |
| 12. Repetitive use of objects |  |  |  | **0.521** |
| 13. Circumscribed interests |  |  |  | **0.369** |
| 14. Unusual sensory interests |  |  |  | **0.848** |
| 15. Hand and finger mannerisms |  | **0.402** |  |  |
| 16. Complex body mannerisms | **0.612** |  |  |  |
| 17. Self-injurious behaviour | **0.555** |  |  |  |
| 18. Unusual attachment to object |  |  |  | **0.580** |
| 19. Friends | **0.612** |  |  |  |
| 20. Social chat |  |  | **0.461** |  |
| 21. Imitation |  |  | **0.661** |  |
| 22. Pointing to express interest | **0.555** |  |  |  |
| 23. Gestures |  | **0.614** |  |  |
| 24. Nodding to say yes | **0.776** |  |  |  |
| 25. Head shaking to mean no | **0.778** |  |  |  |
| 26. Eye gaze |  | **0.547** |  |  |
| 27. Social smiling | **0.474** |  |  |  |
| 28. Showing and directing attention |  |  | **0.722** |  |
| 29. Offering to share | **0.654** |  |  |  |
| 30. Seeking to share enjoyment | **0.634** |  |  |  |
| 31. Offering comfort | **0.370** |  |  |  |
| 32. Quality of social overtures |  |  | **0.609** |  |
| 33. Range of facial expressions |  |  | **0.700** |  |
| 34. Imitative social play | **0.809** |  |  |  |
| 35. Imaginative play | **0.799** |  |  |  |
| 36. Interest in children | **0.681** |  |  |  |
| 37. Response to other children | **0.671** |  |  |  |
| 38. Attention to voice | **0.537** |  |  |  |
| 39. Imaginative play with peers | **0.748** |  |  |  |
| 40. Group play | **0.844** |  |  |  |

*Supplementary Table 6: Factor loadings for the four factors (from the EFA done on the study sample) confirmatory factor analysis model*

Factor loadings for the 39 items in the SCQ; items highlighted in green indicate items with a factor loading threshold of above 0.300 recommended cut-off point and items in red are below this threshold.

|  | **All participants** | | | | **Autism cases** | | | | | **NDD cases** | | | |
| --- | --- | --- | --- | --- | --- | --- | --- | --- | --- | --- | --- | --- | --- |
| **SCQ Items** | **S_X2** | **df s_x2** | **RMSEA** | **pS_X2** | **S_X2** | **df s_x2** | **RMSEA** | **pS_X2** | **S_X2** | | **df s_x2** | **RMSEA** | **pS_X2** |
| 2. Conversation | 12.504 | 12.000 | 0.013 | 0.406 | 6.135 | 5.000 | 0.054 | 0.293 | 6.236 | | 7.000 | 0.000 | 0.513 |
| 3. Stereotyped utterances | 20.857 | 17.000 | 0.029 | 0.233 | 15.621 | 5.000 | 0.166 | 0.008 | 11.879 | | 12.000 | 0.000 | 0.455 |
| 4. Inappropriate questions | 22.136 | 16.000 | 0.038 | 0.139 | 15.336 | 8.000 | 0.109 | 0.053 | 18.063 | | 11.000 | 0.085 | 0.080 |
| 5. Pronoun reversal | 29.700 | 19.000 | 0.046 | 0.056 | 20.625 | 7.000 | 0.159 | 0.004 | 14.741 | | 12.000 | 0.051 | 0.256 |
| 6. Neologisms | 22.847 | 15.000 | 0.044 | 0.087 | 8.661 | 7.000 | 0.056 | 0.278 | 17.475 | | 10.000 | 0.092 | 0.065 |
| 7. Verbal rituals | 14.534 | 14.000 | 0.012 | 0.411 | 3.897 | 5.000 | 0.000 | 0.564 | 10.443 | | 12.000 | 0.000 | 0.577 |
| 8. Compulsions and rituals | 13.935 | 16.000 | 0.000 | 0.604 | 3.694 | 9.000 | 0.000 | 0.930 | 19.484 | | 10.000 | 0.104 | 0.035 |
| 9. Inappropriate facial expressions | 14.607 | 10.000 | 0.042 | 0.147 | 2.080 | 2.000 | 0.023 | 0.353 | 6.790 | | 4.000 | 0.089 | 0.147 |
| 10. Use of other’s body | 16.201 | 12.000 | 0.036 | 0.182 | 13.172 | 10.000 | 0.064 | 0.214 | NA | | NA | NA | NA |
| 11. Unusual preoccupations | 15.179 | 23.000 | 0.000 | 0.888 | 7.040 | 11.000 | 0.000 | 0.796 | 8.683 | | 13.000 | 0.000 | 0.796 |
| 12. Repetitive use of objects | 28.943 | 25.000 | 0.024 | 0.266 | 12.133 | 13.000 | 0.000 | 0.517 | 14.952 | | 11.000 | 0.064 | 0.185 |
| 13. Circumscribed interests | 40.287 | 27.000 | 0.043 | 0.048 | 26.374 | 13.000 | 0.116 | 0.015 | 12.299 | | 13.000 | 0.000 | 0.503 |
| 14. Unusual sensory interests | 19.337 | 20.000 | 0.000 | 0.500 | 10.950 | 10.000 | 0.035 | 0.361 | 8.287 | | 11.000 | 0.000 | 0.687 |
| 15. Hand and finger mannerisms | 22.784 | 18.000 | 0.032 | 0.199 | 11.090 | 11.000 | 0.010 | 0.436 | 4.025 | | 6.000 | 0.000 | 0.673 |
| 16. Complex body mannerisms | 6.048 | 13.000 | 0.000 | 0.944 | 11.028 | 10.000 | 0.037 | 0.355 | 0.005 | | 1.000 | 0.000 | 0.942 |
| 17. Self-injurious behaviour | 19.817 | 15.000 | 0.035 | 0.179 | 15.474 | 12.000 | 0.061 | 0.217 | NaN | | 0.000 | NaN | NaN |
| 18. Unusual attachment to object | 27.086 | 24.000 | 0.022 | 0.300 | 8.196 | 13.000 | 0.000 | 0.831 | 8.952 | | 10.000 | 0.000 | 0.537 |
| 19. Friends | 21.347 | 27.000 | 0.000 | 0.770 | 13.510 | 10.000 | 0.068 | 0.197 | 12.477 | | 14.000 | 0.000 | 0.568 |
| 20. Social chat | 17.709 | 17.000 | 0.012 | 0.407 | 7.310 | 3.000 | 0.137 | 0.063 | 4.704 | | 7.000 | 0.000 | 0.696 |
| 21. Imitation | 29.405 | 22.000 | 0.036 | 0.134 | 14.486 | 12.000 | 0.052 | 0.271 | 12.683 | | 6.000 | 0.113 | 0.048 |
| 22. Pointing to express interest | 27.339 | 24.000 | 0.023 | 0.289 | 7.306 | 8.000 | 0.000 | 0.504 | 14.769 | | 13.000 | 0.039 | 0.322 |
| 23. Gestures | 23.698 | 21.000 | 0.022 | 0.308 | 19.033 | 11.000 | 0.097 | 0.061 | 8.446 | | 10.000 | 0.000 | 0.585 |
| 24. Nodding to say yes | 13.895 | 11.000 | 0.031 | 0.239 | 5.184 | 5.000 | 0.022 | 0.394 | 4.140 | | 7.000 | 0.000 | 0.763 |
| 25. Head shaking to mean no | 18.099 | 11.000 | 0.049 | 0.079 | 7.698 | 4.000 | 0.110 | 0.103 | 6.519 | | 8.000 | 0.000 | 0.589 |
| 26. Eye gaze | 27.759 | 22.000 | 0.031 | 0.184 | 14.238 | 12.000 | 0.049 | 0.286 | 16.341 | | 10.000 | 0.085 | 0.090 |
| 27. Social smiling | 8.408 | 12.000 | 0.000 | 0.753 | 8.327 | 7.000 | 0.050 | 0.305 | 0.908 | | 2.000 | 0.000 | 0.635 |
| 28. Showing and directing attention | 18.981 | 18.000 | 0.014 | 0.393 | 12.023 | 8.000 | 0.081 | 0.150 | 14.025 | | 7.000 | 0.107 | 0.051 |
| 29. Offering to share | 22.920 | 20.000 | 0.023 | 0.293 | 14.783 | 7.000 | 0.120 | 0.039 | 6.038 | | 9.000 | 0.000 | 0.736 |
| 30. Seeking to share enjoyment | 26.257 | 20.000 | 0.034 | 0.157 | 18.128 | 10.000 | 0.103 | 0.053 | 7.196 | | 7.000 | 0.018 | 0.409 |
| 31. Offering comfort | 24.696 | 23.000 | 0.017 | 0.366 | 16.254 | 7.000 | 0.131 | 0.023 | 12.141 | | 12.000 | 0.012 | 0.434 |
| 32. Quality of social overtures | 19.613 | 15.000 | 0.034 | 0.187 | 10.253 | 9.000 | 0.043 | 0.330 | 1.159 | | 2.000 | 0.000 | 0.560 |
| 33. Range of facial expressions | 17.553 | 18.000 | 0.000 | 0.485 | 6.050 | 10.000 | 0.000 | 0.811 | 16.283 | | 6.000 | 0.140 | 0.012 |
| 34. Imitative social play | 24.393 | 13.000 | 0.057 | 0.028 | 15.305 | 2.000 | 0.294 | 0.000 | 10.341 | | 6.000 | 0.091 | 0.111 |
| 35. Imaginative play | 15.262 | 12.000 | 0.032 | 0.227 | 5.302 | 1.000 | 0.236 | 0.021 | 13.461 | | 7.000 | 0.102 | 0.062 |
| 36. Interest in children | 31.338 | 20.000 | 0.046 | 0.051 | 8.291 | 6.000 | 0.070 | 0.218 | 1.271 | | 6.000 | 0.000 | 0.973 |
| 37. Response to other children | 26.904 | 19.000 | 0.039 | 0.107 | 2.331 | 5.000 | 0.000 | 0.802 | 2.151 | | 6.000 | 0.000 | 0.905 |
| 38. Attention to voice | 23.076 | 22.000 | 0.014 | 0.397 | 13.005 | 11.000 | 0.049 | 0.293 | 1.485 | | 4.000 | 0.000 | 0.829 |
| 39. Imaginative play with peers | 15.787 | 14.000 | 0.022 | 0.327 | NaN | 0.000 | NaN | NaN | 11.592 | | 9.000 | 0.057 | 0.237 |
| 40. Group play | 8.785 | 12.000 | 0.000 | 0.721 | NaN | 0.000 | NaN | NaN | 3.464 | | 7.000 | 0.000 | 0.839 |

*Supplementary Table 7: Item response theory degree fit of SCQ items based on three groups, the entire sample, autism cases and cases with an NDD diagnosis*; S-X² - item fit chi-square based statistic used to assess how well an item test fits the assumptions of the item response theory (IRT) model being used; df -degrees of freedom; RMSEA - Root Mean Square Error of Approximation (RMSEA), lower RMSEA values indicate a better fit; pS_X2 p-value based on the item fit chi-square based statistic

|  | **All participants** | | **Autism cases** | | **NDD cases** | |
| --- | --- | --- | --- | --- | --- | --- |
| **Items** | **Discrimination parameter** | **Difficulty parameter** | **Discrimination parameter** | **Difficulty parameter** | **Discrimination parameter** | **Difficulty parameter** |
| 2. Conversation | 4.075 | 0.643 | 2.427 | -0.792 | 1.342 | 1.288 |
| 3. Stereotyped utterances | 2.795 | 0.453 | 2.401 | -0.945 | 0.604 | 1.217 |
| 4. Inappropriate questions | 2.542 | 0.759 | 1.660 | -0.512 | 0.459 | 2.405 |
| 5. Pronoun reversal | 2.306 | 0.439 | 1.970 | -0.757 | 0.549 | 0.668 |
| 6. Neologisms | 2.813 | 0.713 | 1.847 | -0.678 | 0.699 | 2.053 |
| 7. Verbal rituals | 3.545 | 0.385 | 2.460 | -0.991 | 0.851 | 0.471 |
| 8. Compulsions and rituals | 2.329 | 0.898 | 1.214 | -0.496 | 0.530 | 3.027 |
| 9. Inappropriate facial expressions | 0.796 | 3.889 | 0.505 | 5.121 | 0.327 | 6.810 |
| 10. Use of other’s body | 3.117 | 1.298 | 1.304 | 0.212 | 1.205 | 3.650 |
| 11. Unusual preoccupations | 1.064 | 0.297 | 0.458 | -1.592 | 0.446 | -0.155 |
| 12. Repetitive use of objects | 1.029 | 1.390 | 0.260 | 0.397 | -0.179 | -6.965 |
| 13. Circumscribed interests | 0.534 | 0.239 | 0.361 | -0.889 | 0.508 | -0.043 |
| 14. Unusual sensory interests | 1.905 | 0.728 | 1.021 | -0.622 | 0.780 | 1.287 |
| 15. Hand and finger mannerisms | 1.261 | 2.010 | 0.146 | 7.337 | 0.990 | 2.201 |
| 16. Complex body mannerisms | 2.814 | 1.348 | 1.363 | 0.363 | 0.372 | 8.391 |
| 17. Self-injurious behaviour | 2.293 | 1.426 | 0.802 | 0.441 | 0.285 | 11.893 |
| 18. Unusual attachment to object | 1.184 | 1.290 | 0.339 | 1.420 | 0.870 | 0.976 |
| 19. Friends | 0.741 | 0.434 | 0.741 | -1.048 | 0.190 | 2.808 |
| 20. Social chat | 1.117 | -0.863 | 1.577 | -1.690 | 1.592 | -0.557 |
| 21. Imitation | 1.762 | 0.767 | 0.888 | -0.202 | 2.547 | 0.424 |
| 22. Pointing to express interest | 1.326 | 0.743 | 1.636 | -0.323 | 0.320 | 2.169 |
| 23. Gestures | 1.922 | 1.061 | 1.003 | 0.064 | 0.765 | 1.815 |
| 24. Nodding to say yes | 3.832 | 0.863 | 3.550 | -0.338 | 1.135 | 1.905 |
| 25. Head shaking to mean no | 3.566 | 0.802 | 3.613 | -0.445 | 0.953 | 1.958 |
| 26. Eye gaze | 1.589 | 1.134 | 0.984 | 0.253 | 0.807 | 1.486 |
| 27. Social smiling | 1.934 | 1.720 | 1.503 | 0.991 | 2.259 | 1.884 |
| 28. Showing and directing attention | 2.092 | 0.821 | 1.576 | -0.092 | 2.227 | 0.630 |
| 29. Offering to share | 1.971 | 0.689 | 1.910 | -0.481 | 1.167 | 1.084 |
| 30. Seeking to share enjoyment | 1.875 | 0.887 | 1.267 | -0.318 | 1.795 | 1.109 |
| 31. Offering comfort | 0.771 | -0.220 | 1.744 | -0.697 | 0.773 | -0.091 |
| 32. Quality of social overtures | 2.223 | 1.463 | 1.355 | 0.746 | 2.448 | 1.564 |
| 33. Range official expressions | 2.272 | 1.119 | 1.447 | 0.300 | 2.031 | 1.103 |
| 34. Imitative social play | 3.687 | 0.626 | 8.259 | -0.435 | 2.328 | 0.739 |
| 35. Imaginative play | 3.745 | 0.288 | 5.965 | -0.807 | 2.135 | 0.115 |
| 36. Interest in children | 2.136 | 0.727 | 2.703 | -0.274 | 2.274 | 0.828 |
| 37. Response to other children | 2.206 | 0.898 | 2.716 | -0.159 | 1.805 | 1.215 |
| 38. Attention to voice | 1.515 | 1.342 | 0.885 | 0.479 | 2.443 | 1.296 |
| 39. Imaginative play with peers | 2.923 | 0.208 | 4.892 | -0.970 | 1.195 | 0.134 |
| 40. Group play | 4.530 | 0.455 | 30.729 | -0.663 | 2.267 | 0.587 |

*Supplementary Table 8: Item difficulty and discrimination Parameters based on the entire sample, autism cases and cases with an NDD diagnosis*
