# Supplementary material for: Evaluation of the Psychometric Properties of the Social Communication Questionnaire in Rural Kenya
Source: J Autism Dev Disord. Author manuscript; Available in PMC 2025 Aug 1. (PMC7616653; doi:10.1007/s10803-024-06380-9)
Supplement: Supplementary file 1 [file EMS198924-supplement-Appendix.pdf]

Appendix 1: The Social Communication Questionnaire Items

Instruction: Answer each question by answering yes or no. A few questions ask about several related types of behaviour; please answer yes if any of these behaviours have ever been present. Although you may be uncertain about whether some behaviours were ever present or not, please answer yes or no to every question on the basis of what you think.

1. Is she/he now able to talk using short phrases or sentences? If no, skip to question 8
2. Can you have a to and fro "conversation with her/him that involves taking turns or building on what you have said?
3. Has she/he ever used odd phrases or said the same thing over and over in almost exactly the same way (either phrases that she /she has heard other people use or ones that she/he has made up)?
4. Has she/he ever used socially inappropriate questions or statements? for example has she/he ever regularly asked personal questions or made personal comments at awkward times?
5. Has she/he ever got her/his pronouns mixed up (E.g., saying you or she/he for I)?
6. Has she/he ever used words that she/he seemed to have invented or made up her/himself, put things in odd, indirect ways, or used metaphorical ways of saying things (e.g., saying hot rain for steam)?
7. Has she/he ever said the same thing over and over in exactly the same way or insisted that you say the same thing over and over again?
8. Has she/he ever had things that she/he seemed to have to do in a very particular way or order or rituals that she/he insisted that you go through?
9. Has her/his facial expression usually seemed appropriate to the particular situation, as far as you could tell?
10. Has she/he ever used your hand like a tool or as if it were part of her/his own body (e.g., pointing with your finger, pitting your hand on a doorknob to get you open the door)?
11. Has she/he ever had any interests that preoccupy her/him and might seem odd to other people (e.g. traffic lights, drainpipes or timetables)?
12. Has she/he ever seemed to be interested in parts of a toy or an object (e.g., spinning the wheels of a car,) rather than using the object as it was intended?
13. Has she/he ever had any special interest that were unusual in their intensity but otherwise appropriate for her/his age and peer group (e.g., trains, dinosaurs)?
14. Has she/he ever seemed to be unusually interested in the sight, feel, sound, taste, or smell of things or people?
15. Has she/he ever had any mannerisms or odd ways of moving her/his hands or fingers, such as flapping or moving her/his fingers in front of her/his eyes?
16. Has she/he ever had any complicated movements of her/his whole body, such as spinning or repeatedly bouncing up and down?
17. Has she/he ever injured her/himself deliberately, such as by biting her his arm or banging her/his head?
18. Has she/he ever had any objects (other than a soft toy or comfort blanket) that she/he had to carry around?
19. Does she/he have any particular friends or a best friend?

For the following behaviours, please focus on the time period between the child's fourth and fifth birthdays. You may find it easier to remember how things were at that time by focusing on key events, such as starting school, moving house, Christmastime, or other specific events that are particularly memorable for you as a family. If your child is not yet 4 years old, please consider her or his behaviour in the past 12 months.

20. When she/he was 4 to 5, did she/he ever talk with you just to be friendly (rather than to get something)?
21. When she/he was 4 to 5, did she/he ever spontaneously copy you (Or other people) or what you were doing (such as vacuuming, gardening or mending things)?

22. When she/he was 4 to 5, did she/he ever spontaneously point at things around her/him just to show you things (not because she/he wanted them)?
23. When she/he was 4 to 5, did she/he ever use gestures other than pointing or pulling your hand, to let you know what she/he wanted? yes no
24. When she was 4 to 5, did she/he nod her/his head to mean yes?
25. When she/he was 4 to 5, did she/he shake his head to mean no?
26. When she/he was 4 to 5, did she/he usually look at you directly in the face when doing things with you or talking with you?
27. When she/he was 4 to 5, did she/he smile back if someone smiled at her/him?
28. When she/he was 4 to 5, did she/he ever show you things that interested her/him to engage your attention?
29. When she/he was 4 to 5, did she/he ever offer to share things other than food with you?
30. When she was 4 to 5, did she/he ever seem to want you to join in her/his enjoyment of something?
31. When she/he was 4 to 5, did she/he ever try to comfort you if you were sad or hurt?
32. When she/he was 4 to 5, when she/he wanted something or wanted help, did she/he look at you and use gestures with sounds or words to get your attention?
33. When she/he was 4 to 5, did she/he show a normal range of facial expressions?
34. When she was 4 to 5, did she/he ever spontaneously join in and try to copy the actions in social games, such as *the mulberry Bush or London Bridge is Falling Down?*
35. When she/he was 4 to 5, did she/he play any pretend or make-believe games?
36. When she/he was 4 to 5, did she/he seem interested with other children of approximately the same age whom she/he did not know?
37. When she/was 4 to 5, did she/he respond positively when another child approached her/him?
38. When she/he was 4 to 5, if you came into a room and started talking to her/him without calling her/his name, did she/he usually look up and pay attention to you?
39. When she/he was 4 to 5, did she/he ever play imaginative games with another child in such a way that you could tell that they each understood what the other was pretending?
40. When she/he was 4 to 5, did she/he play cooperatively in games that required joining in with a group of other children, such as hide-and-seek or ball games?
